# All-Atom Simulations Reveal the Effect of Membrane Composition on the Signaling of the NKG2A/CD94/HLA-E Immune Receptor Complex

**DOI:** 10.1021/acs.jcim.4c01357

**Published:** 2024-12-02

**Authors:** Martin Ljubič, Andrej Perdih, Jure Borišek

**Affiliations:** † National Institute of Chemistry, Hajdrihova 19, 1000 Ljubljana, Slovenia; ‡ Faculty of Pharmacy, University of Ljubljana, Aškerčeva 7, 1000 Ljubljana, Slovenia

## Abstract

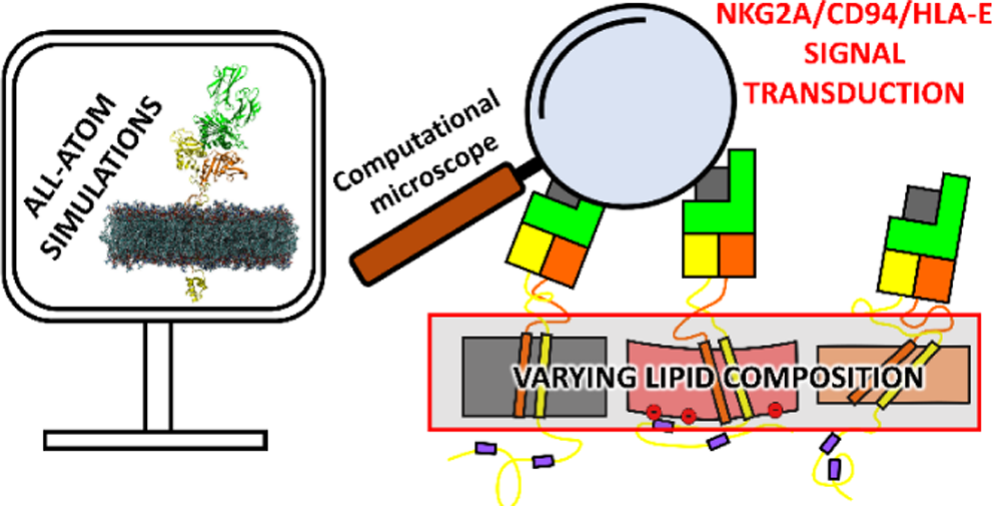

Understanding how membrane composition influences the dynamics and function of transmembrane proteins is crucial for the comprehensive elucidation of cellular signaling mechanisms and the development of targeted therapeutics. In this study, we employed all-atom molecular dynamics simulations to investigate the impact of different membrane compositions on the conformational dynamics of the NKG2A/CD94/HLA-E immune receptor complex, a key negative regulator of natural killer cell cytotoxic activity. Our results reveal significant variations in the behavior of the immune complex structure across five different membrane compositions, which include POPC, POPA, DPPC, and DLPC phospholipids, and a mixed POPC/cholesterol system. These variations are particularly evident in the intracellular domain of NKG2A, manifested as changes in mobility, tyrosine exposure, and interdomain communication. Additionally, we found that a large concentration of negative charge at the surface of the POPA-based membrane greatly increased the number of contacts with lipid molecules and significantly decreased the exposure of intracellular NKG2A ITIM regions to water molecules, thus likely halting the signal transduction process. Furthermore, the DPPC model with a membrane possessing a high transition temperature in a gel-like state became curved, affecting the exposure of one ITIM region. The decreased membrane thickness in the DPLC model caused a significant transmembrane domain tilt, altering the linker protrusion angle and potentially disrupting the hydrogen bonding network in the extracellular domain. Overall, our findings highlight the importance of considering membrane composition in the analysis of transmembrane protein dynamics and in the exploration of novel strategies for the external modulation of their signaling pathways.

## Introduction

In recent years, considerable efforts have been made to understand how the immune system works, with the aim of identifying new therapeutic avenues to eliminate tumors and senescent cells that accumulate during many age-related diseases.^1^ Recent developments have in part focused on studying natural killer (NK) cells, which play an important role in the innate immune response against a variety of pathogens and in the regulation of the immune system, as well as in the production of cytokines such as interferon γ (IFN-γ).^2^ Despite significant advances, much remains to be unraveled about the complex events governing cell signaling via these systems, with probably one of the least explored aspects being the effect of membrane composition on the immune cell signaling, particularly at an atomistic level.

On their surface, the NK cells harbor several activating and inhibitory transmembrane receptors that control their cytotoxic activity. Inhibitory NK receptors embedded in the membrane of the immune cell [e.g., killer immunoglobulin-like receptors, Ig-like receptors, leukocyte inhibitory receptors, and C-type lectin receptors (NKG2A-CD94)] are crucial for preventing attacks on healthy cells.^3^ Healthy cells express HLA class I ligands with a high degree of polymorphism as well as structurally conserved nonclassical ligands such as human leukocyte antigen-E (HLA-E), which is present in a trimeric complex of light and heavy chains, β-2m, and a nonameric peptide, affecting protein binding and recognition.^4^ HLA-E binds specifically to the NKG2A/CD94 complex, a dimeric C-type lectin receptor embedded in the cell membranes of NK cells. This receptor is associated with pathologies such as cancer and could be important in the development of age-related diseases, which are associated with an increased amount of senescent cells. They all exhibit upregulation of HLA-E and are therefore able to evade elimination by inhibiting NK cell cytotoxicity.^5^ Therapeutically blocking the NKG2A/CD94-HLA-E interaction has so far been successfully used to enhance the immune response of NK cells against tumors.^6,7^

Membranes in which the inhibitory receptors reside can effectively tune these physiological processes by providing a platform to either enhance or diminish the intensity of the signaling cascades and consequently immune responses against attacks from pathogens or tumor cells.^8^ Inhibition of NK cell action via the NKG2A/CD94 receptor involves a cytoplasmic Immunoreceptor Tyrosine-based Inhibition Motif (ITIM).^9^ Phosphorylation of ITIMs by Src kinases triggers subsequent recruitment of SH2 domains of SHP phosphatases, ultimately leading to the dephosphorylation of key signaling molecules (e.g., Vav1) involved in NK cell activation.^5^ One of the suggested mechanisms for the ITIM-bearing immune receptor inactivation involves interactions between the membrane phospholipids and the mobile NKG2A intracellular region, which is capable of forming dynamic α-helices.^10^ Lipid molecules have also been discovered to bind SH2, possessing a regulatory role in protein–protein interactions and downstream signaling.^11^ Differences in the composition of the membrane have been shown to significantly impact the cytotoxic activity of immune cells,^12^ warranting further investigation into how membrane composition affects the NK or T cell’s ability to maintain its cytotoxic activity.

Since the membrane environment of cells is diverse and dynamic, accurate replication in experimental settings poses numerous challenges. Considerable efforts have been made to increase the scope and complexity of the models used in these studies.^13^ With the advent of modern computational chemistry, it has become possible to systematically study the effects of membrane composition on the protein structure and dynamics at the atomistic level by investigating phenomena such as lipid interactions.^14,15^ This is especially relevant in the case of transmembrane receptors where a large portion of their structure is imbedded in the membrane However, so far, little attention has been devoted to studying the intricate effects of a changing membrane composition,^16^ which could lead to a skewed picture of the understanding of membrane systems and the proposed mechanism of action.

The key events of signal transduction by the inhibitory NKG2A/CD94 receptor have been computationally examined via comparison of the NKG2A/CD94 receptor structure with the HLA-E bound complex in a standard **POPC** membrane.^17^ To extend our knowledge of the behavior of these inhibitory receptors and potentially transmembrane proteins even more broadly, we have now investigated the key effects of the membrane lipid composition on the dynamics and signal transduction of the immune complex NKG2A/CD94/HLA-E in all-atom molecular dynamics (MD) simulations under exaggerated membrane conditions. We assessed key geometrical parameters of the extracellular (ECD), transmembrane (TMD), and intracellular (ICD) domains of this receptor as well as its global dynamics to better understand the effects of five drastically different membrane types on the complex involvement in the initial step of the inhibitory signaling cascade.

Currently, the research that studies the influence of lipid composition on the conformation and function of the embedded large protein systems is in its early stages.^16,18,19^ In this respect, our study represents another systematic computational evaluation of the often-overlooked complex nature of cell membrane parameters and their influence on transmembrane receptor signaling. The results could guide future experiments to better assess these effects and aid in designing potential therapeutic interventions to treat various diseases such as cancer and age-related conditions associated with the accumulation of senescent cells.

## Methods

### Structural Models of the Complete Immune NKG2A/CD94 Receptor and Its Complex with HLA-E

The structural model of the NKG2A/CD94/HLA-E complex was constructed as described before.^17^ The system was constructed from the experimentally determined structure of the extracellular NKG2A/CD94/HLA-E domains, solved at a 3.4 Å resolution (PDB ID: 3CDG),^20^ with the missing transmembrane and intracellular parts being built using Alphafold 2.^21,22^

**Figure 1:**
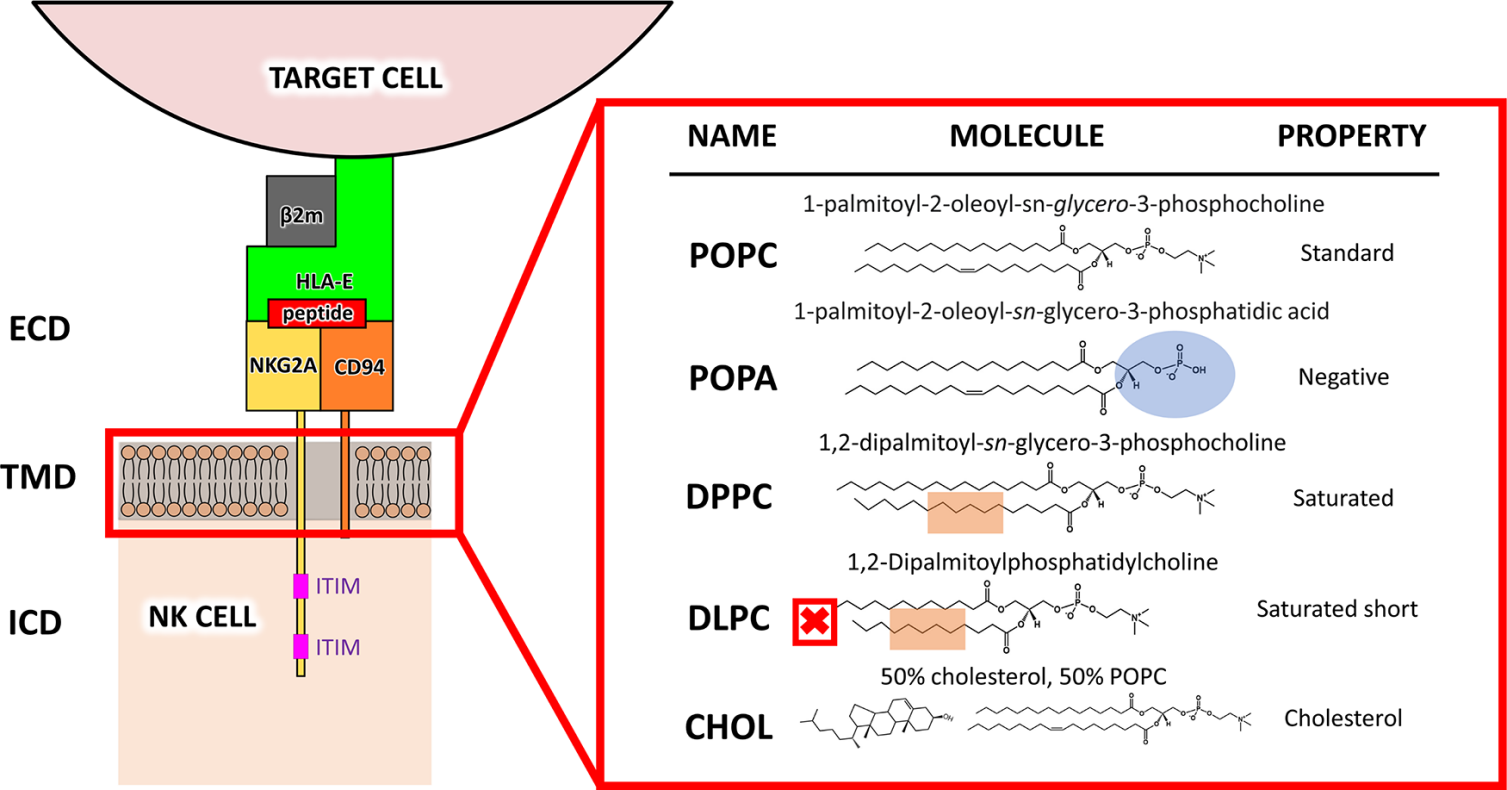
Schematic representation of the inhibitory NKG2A/CD94/HLA-E complex embedded in one of several types of membranes, with used phospholipids and their most important properties on the right side (from top to bottom: **POPC**, **POPA**, **DPPC**, **DLPC**, and the mixed **CHOL** model). NKG2A (yellow) and CD94 (orange) proteins form a dimeric transmembrane receptor located on the surface of NK cells. The intracellular (ICD) ITIM regions are phosphorylated upon activation of the receptor, which occurs when HLA-E (green), which is overexpressed on senescent or cancer cells, binds to the receptor in the extracellular region (ECD). Since the receptor transmembrane domain (TMD) is embedded in the NK cell membrane, changes in membrane composition can influence protein dynamics and signal transduction.

We constructed membranes comprised of **POPC**, **POPA**, **DPPC**, and **DLPC** phospholipids using MemGen^23^ with 225 phospholipids per monolayer and a 65 Å^2^ area per phospholipid. Each membrane has a distinct property, as outlined in Figure 1. Additionally, we built a **POPC**/**cholesterol** mixed membrane, hereafter referred as **CHOL**, in which the cholesterol was evenly distributed in the **POPC** bilayer at 50% (Figure 1). Next, we manually inserted the protein structures in the lipid bilayer using ChimeraX^24^ by aligning the protein structure with the generated membrane. The entire structure was aligned so that the NKG2A and CD94 helixes were positioned at the centers of the membranes to match the middle of the hydrophobic regions of the bilayers. The helices were positioned perpendicular to the plane of the membrane to ensure consistency across all membrane models. We created a 3 Å hole around the protein structure in the **POPC**, **POPA**, and **CHOL** membranes and a 1.5 Å hole in the **DPPC** and **DLPC** membranes. The smaller sizes of the holes of the latter two systems were used to ensure that the systems could properly equilibrate as membrane stability issues were present in these systems when using a 3 Å hole.

### Molecular Dynamics Simulations

Classical molecular dynamics (MD) simulations were conducted using Amber20 PMEMD software package.^25^ The AMBER-ff19SB force field (FF) was employed for modeling proteins, while the Lipid17 force field was utilized to describe the membrane components.^26,27^ The protonation states of the ionizable residues were determined at a neutral pH condition of 7 with the PDB 2PQR web tool.^28^ Carboxylic amino acids were observed in their typical deprotonated states, while histidines were found to be protonated at Nε, Nδ, or both positions.

The protein complex–membrane systems were solvated using Gromacs 2019,^29^ incorporating TIP3P water molecules to match the size of the lipid membranes, resulting in a box dimension of roughly 120 × 120 × 320 Å^3^. Water molecules within the membrane were subsequently eliminated, and each generated system, including Na^+^ counterions and water molecules, amounted to between 400,000 and 500,000 atoms. Disulfide bonds were constructed using the *tleap* module of Ambertools20,^25^ which was also employed for preparing the topologies of the models.

The systems underwent an initial two-step minimization employing a steepest descent algorithm, followed by a conjugate gradient algorithm. Subsequent to gradual heating to 300 K in a single step over 300 ps with positional restraints of 100 kcal/mol Å^2^ on heavy atoms, restraints were removed. Five steps of 1 ns simulations in the isothermal–isobaric ensemble (NPT) function were performed, achieving pressure control (1 bar) using a Berendsen barostat^30^ to equilibrate the membrane lipids and periodic boundary conditions. The *skinnb* value was increased to 5 during this step to avoid errors. Productive MD was then carried out in the NPT ensemble. The duration of each simulation was 2 μs. Two additional shorter replicas were simulated for each studied system, lasting up to 1.2 us.

During the MD simulations, temperature control (300 K) was maintained using the Langevin thermostat^31^ with a collision frequency of 1 ps^–1^. The SHAKE algorithm^32^ was applied to constrain bonds involving hydrogen atoms and heavy atoms, and the particle mesh Ewald method^33^ with a cutoff of 10 Å addressed long-range electrostatic interactions. An integration time step of 2 fs was employed throughout all of the MD runs.

### Analysis of Simulation Trajectories

For the visualization and examination of the obtained molecular trajectories, Visual Molecular Dynamics (VMD) software package (VMD),^34^ PyMol,^35^ and ChimeraX^24^ were used. Trajectory analyses, such as root-mean-square fluctuations (RMSF) and cross-correlation matrices, were conducted with *cpptraj*(36) module in Ambertools20 on the equilibrated portion of the stripped trajectories, excluding water and counterions. The initial 500 ns of trajectories were omitted to ensure a comprehensive sampling of the generated conformational space when the system was fully equilibrated. Identical methodology was applied when analyzing the two additional replica simulations of each system. The results of the replicas are presented and discussed separately at the end of the discussion section as well as in the Supporting Information.

Clustering of conformations was performed in *cpptraj* using a hierarchical agglomerative approach with an epsilon value of 7 and a distance metric of root-mean-square deviation (RMSD) of backbone protein atoms.

*Cpptraj* was used to calculate electron density profiles, area per lipid values, and lipid order parameters for the lipid bilayers and to calculate radial distribution functions (RDF) for water molecules surrounding defined protein atoms. It was further employed to assess the extent of receptor head tilting in response to ligand binding, by defining two vectors – vertical and horizontal – to describe the rotation angles. The angles between vector pairs were determined from the vector dot product with the unit cell vector. The analysis of secondary structure changes throughout the simulation was carried out with the DSSP algorithm.^37^

### Cross-Correlation Matrices and Correlation Scores

The cross-correlation matrices, employing Pearson’s correlation coefficients (CC_*ij*_), served to quantify the correlated and anticorrelated motions between residue pairs along the generated molecular trajectory. The CC_*ij*_ values range from −1 (entirely anticorrelated motion) to +1 (fully correlated motion), with 0 signifying no correlation. The Pearson correlation coefficient (*C*_*ij*_) is computed on the Cα atom pairs, *i* and *j*, according to eq 1
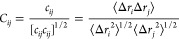
1Here, *c_ij_* is defined as *c*_*ij*_ = ⟨Δ*r*_*i*_Δ*r*_*j*_⟩, Δ*r_i_* represents the displacement vector of atom *i*, and Δ*r_j_* of atom *j*, with the brackets indicating an ensemble average.

Initially, covariance matrices were constructed from the atom position vectors utilizing Cα atoms of the protein backbone. To exclusively capture the internal dynamics of the complex, RMS-fit to a reference structure (an averaged structure from each MD run) was executed, eliminating rotational and translational motions as previously outlined.^38−40^ Subsequently, cross-correlation matrices (normalized covariance matrices) were computed from the covariance matrices using the *cpptraj* module of Ambertools20.^25^

For a simplified representation of relationships within the NKG2A/CD94/HLA-E immune complex, correlations for each protein/domain pair were evaluated by summing the correlation scores (CSs) between each protein/domain and all others. Furthermore, a correlation density for each region was derived by summing the CSs of the protein–domain pair and dividing the result by the product of the number of residues belonging to that pair of proteins/domains. This process yielded a simplified variant of the CC_*ij*_ matrices.

### Principal Component Analysis

Principal component analysis (PCA)^41^ was performed using *cpptraj* to unveil the essential dynamics of proteins.^42^ The process began with the generation of mass-weighted covariance matrices for the Cα atoms. These matrices were constructed from the position vectors of the atoms following an RMS-fit to the reference starting configuration of the MD production run, effectively eliminating rotational and translational motions. The eigenvectors corresponding to the largest eigenvalues were identified as principal components (PCs), signifying the directions of the most relevant motions sampled during the simulation. By projecting the displacement vectors of each atom along these eigenvectors, dimensionality and noise in the trajectory were reduced, revealing only the most significant motions. The cumulative variance accounted for by the PCs was computed for all three models using Gromacs 19.^29^ Essential dynamics along the principal eigenvectors were visualized with Normal Mode Wizard plugin^41^ within the VMD program,^34^ and arrows were drawn to highlight their direction.

## Results

We inserted the NKG2A/CD94/HLA-E immune complex into the membranes composed of phospholipids **POPC**, **POPA**, **DLPC**, and **DPPC** having diverse properties, in addition to the mixed **POPC** phospholipid/cholesterol **CHOL** system (Figure 1) and performed 2 μs classical molecular dynamics (MD) simulations of all systems (see Methods). Simulation trajectories and some results for the **POPC** system that are here used for comparison were taken from a previous work.^17^

### Visual Analysis Reveals That Membrane Composition Affects the Protein Behavior in All Three Protein Domains

**Figure 2:**
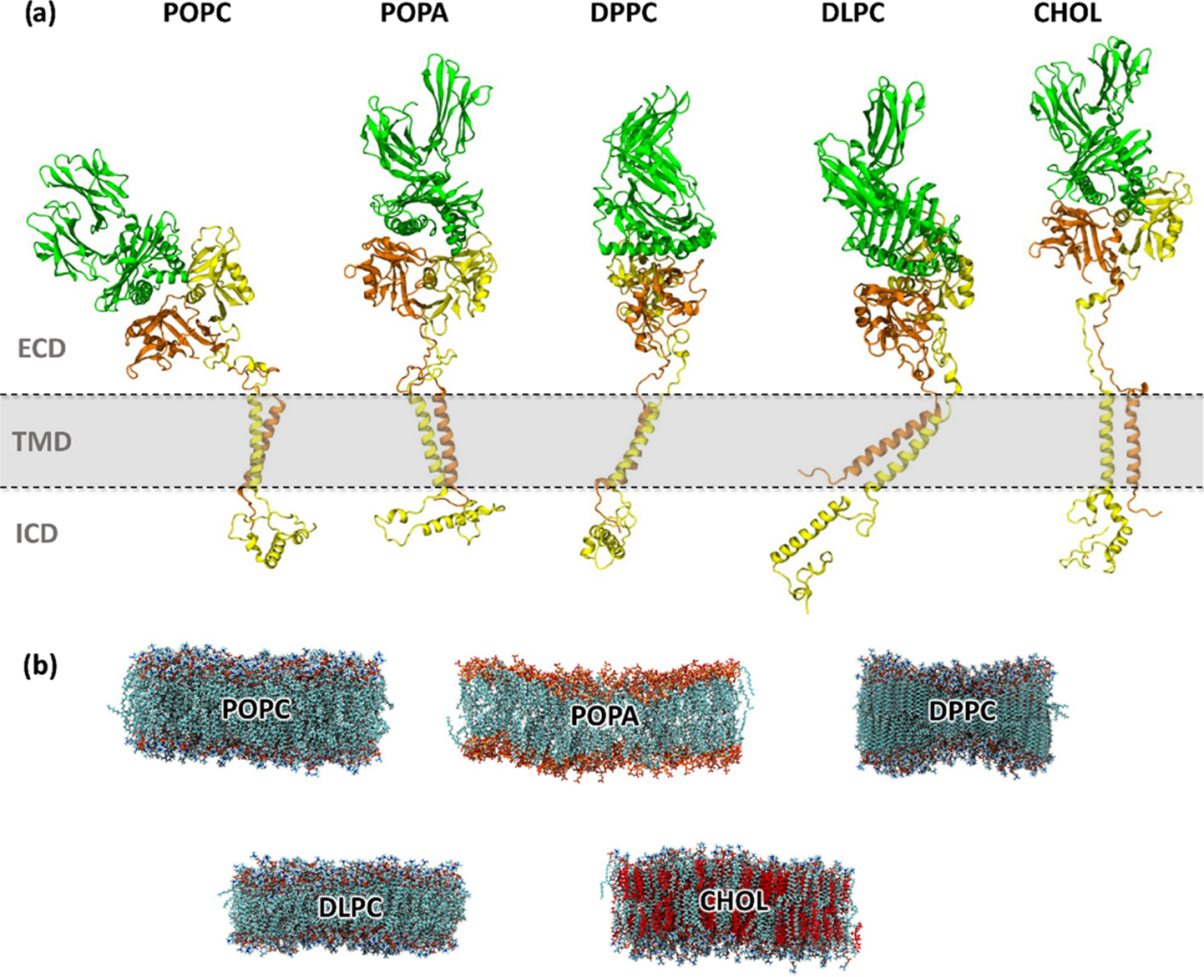
Structural representations of (a) NKG2A/CD94/HLA-E protein complexes and (b) membranes of simulated systems, comprised of **POPC**, **POPA**, **DPPC**, **DLPC**, and **CHOL**. The images of the protein structures were generated using hierarchical clustering, and the last simulation frame was represented in membrane-only representations.

Initially, we performed hierarchical clustering of the NKG2A/CD94/HLA-E complex trajectories to better visualize the most representative protein conformations of each system. Significant visual differences were observed in the extracellular domain (ECD), transmembrane domain (TMD), and intracellular domain (ICD) of the NKG2A/CD94/HLA-E complex (Figure 2a). When the standard **POPC** membrane was used, the NKG2A/CD94 receptor head resided relatively close to the membrane during a significant part of the simulation time. However, it retained its flexibility and also displayed upright conformations of the receptor head during the latter half of the simulation.^17^**POPA** with its negatively charged surface was different in the ICD region to **POPC**, with the IC domain of NKG2A protein being quite static and residing at the inner membrane. The saturated **DPPC** model did not display large abnormalities in the protein structure; however, differences in the overall shape of the membrane were observed, potentially affecting the protein ICD (Figure 2b). **DLPC** had a striking difference in TMD positioning, with both TMD tilted to the side to accommodate a smaller membrane size due to the smaller lipids that comprise it. Lastly, the mixed **CHOL** system showed the linker regions in an elongated shape in the cholesterol-rich membrane, pushing the ECD away from the membrane.

Some of these differences in the protein conformations were influenced by the membrane lipid composition, resulting in different membrane dynamics, size, and shape (Figure 2b). Membrane thickness was a major point of difference between models, especially in the saturated short **DLPC** system, in which the bilayer was thinner. The normalized electron density profiles revealed here the difference between the two electron density peaks, corresponding to a thickness of 30 Å (Figure S1). This distance was increased to 37 Å in standard **POPC**, illustrating the expected increase in the membrane thickness in membranes composed of longer lipids. In addition, the peaks were higher in **POPA** because its lipid head groups are negatively charged and create a stronger charge difference between the outer and inner membrane leaflets. The second saturated **DPPC** system notably had broader peaks due to the observed curvature of the membrane, which affected the electron density calculations. Visual inspection of the membranes confirms these findings (Figure 2b), with **DPPC** curvature indicating that the membrane is in a gel form at this temperature. Such behavior has already been reported in the literature.^43,44^

Area per lipid values indicated that the membranes were equilibrated very early into the production run of the simulations and then remained stable (Figure S2). Differences were noted between models, which roughly conform to reported values in other works.^45,46^ The effect of cholesterol on the **POPC** membrane was shown to result in a significant decrease in the area per lipid value. We also calculated order parameters S^CD^ for lipids in the membranes that showed **DPPC**, **CHOL**, and **POPA** displaying the highest absolute values (Figure S3). The ordering effect of cholesterol was seen in a −0.1 increase in S^CD^ from the standard **POPC**. The values in **DPPC** almost reached −0.4 due to the gel-like state and curvature effects, similar to experimental values, and negative **POPA** membrane has also been shown to have higher values.^47,48^**DLPC** had the lowest values, likely in part due to the perturbing effect of the membrane spanning TMD, which the bilayer was forced to accommodate due to a hydrophobic mismatch. Overall, we can discern that the membrane composition significantly changed the properties such as thickness, charge, and criticality, which have likely affected the behavior of the protein in its positioning and mobility.

### Membrane Composition Affects Protein Flexibility and Communication Between Its Domains

Root-mean-square deviation (RMSD) analyses revealed that the values for the negatively charged **POPA** membrane were the highest at 28 Å (Figure S4a), which indicates the largest deviation from the initial structure. This RMSD value was, however, very consistent compared to the other models, confirming the visual observations that in this simulation, the ICD stuck to the membrane surface due to strong electrostatic interactions between the negatively charged lipid heads and the positively charged protein residues and stayed in this position during the simulation. The other models averaged lower RMSDs of 20 Å, with the standard **POPC** composition exhibiting the greatest fluctuations across its trajectory, also observed through visual inspection (Figure S4b).^17^

**Figure 3:**
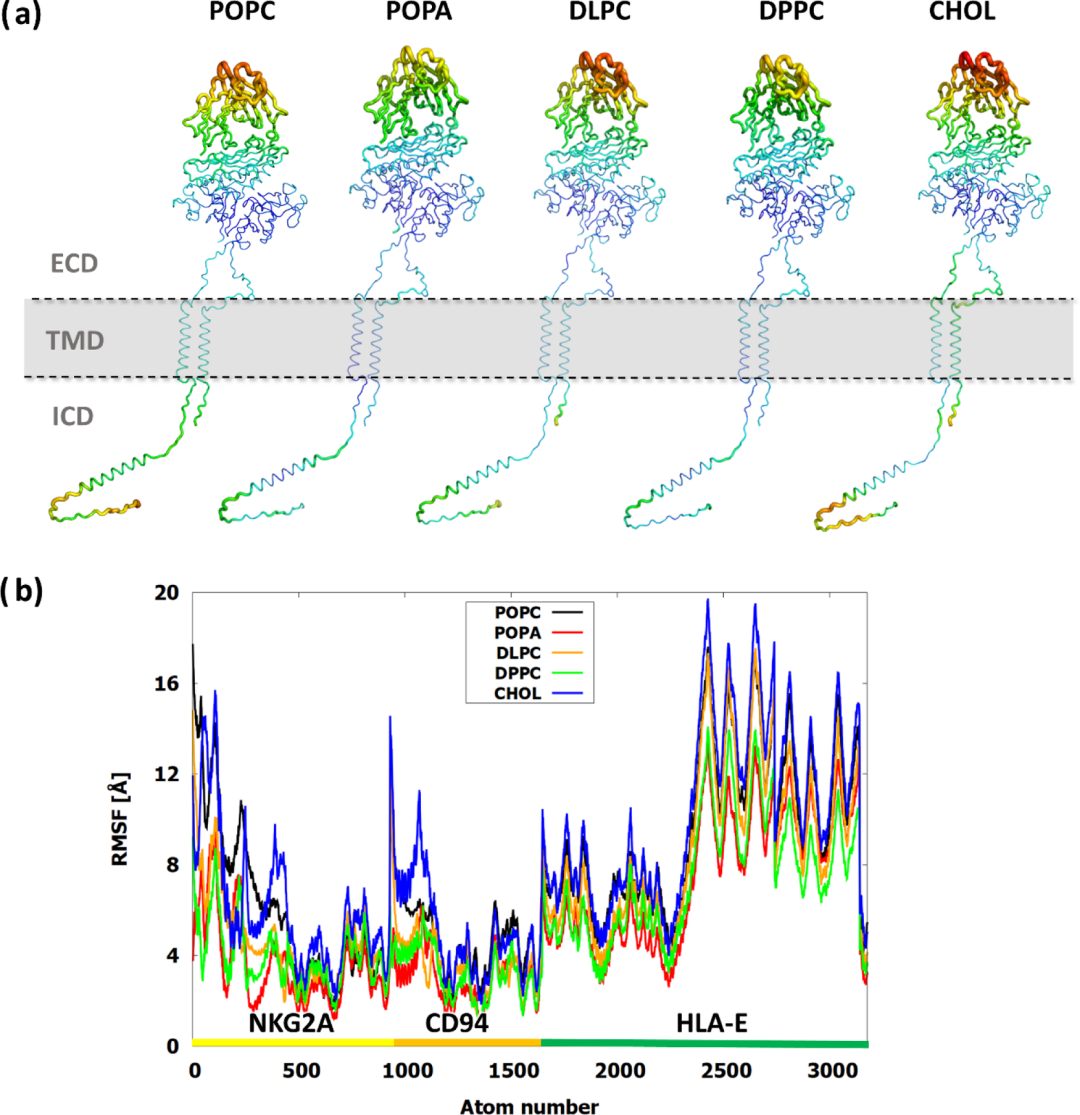
Flexibility of the protein domains of the NKG2A/CD94/HLA-E complex depicted as (a) B-factor representations, projected onto the same starting structure of the complex and (b) RMSF plot for the **POPC, POPA, DLPC, DPPC**, and **CHOL** models. Only the receptor structure was used in the alignment process.

Root-mean square fluctuations (RMSF) can better assess the local flexibility of the protein complex and its domains during the simulation (Figure 3). The negative **POPA** membrane consistently exhibited the smallest RMSF values, followed by the saturated **DPPC** system. In contrast, the **CHOL** and **POPC** membranes showed the highest flexibility. This was especially visible in the IC region of the complex where standard **POPC** had slightly higher RMSF values, but for **CHOL** the values were much higher in the TM and linker region. The RMSF values were also lower in the HLA-E region of **POPA** and **DPPC**, indicating a more static ECD structure in the negative and gel-like membranes.

**Figure 4:**
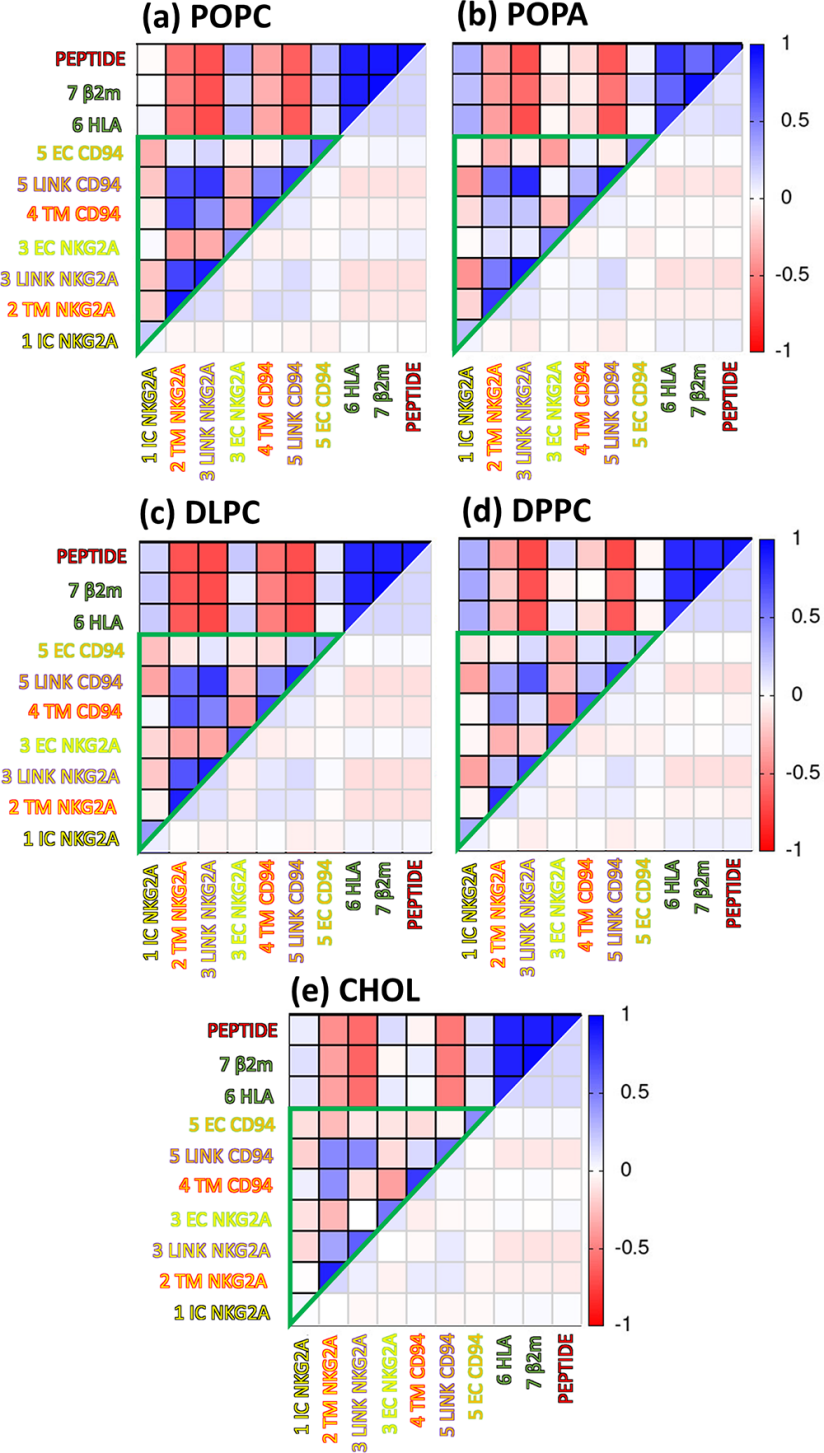
Simplified cross-correlation matrices for the NKG2A/CD94 protein inserted in membrane models (a) **POPC**,^17^ (b) **POPA**, (c) **DLPC**, (d) **DPPC**, and (e) **CHOL**. All frames were aligned to the receptor structure during the correlation matrix calculations.

Next, dynamic cross-correlation (CC) matrices were calculated to investigate the correlations between different protein domains (Figure S5a). Simplified matrices were calculated to better help visualize correlations between domains (Figure 4, numerical values in Figure S5b). The simplified CC matrix of the complex in the standard **POPC** membrane showed strong correlations between linker and TMD regions of NKG2A and CD94 protein (0.4–0.95), with anticorrelations occurring between the ECD of NKG2A and TM regions together with linkers (−0.3) and moderate anticorrelations between ICD NKG2A and other receptor domains (0 to −0.3).^17^ In the case of the saturated short **DLPC** lipids, the simplified CC matrix was very similar and showed the same trends and intensity. On the other hand, the saturated **DPPC** and mixed **CHOL** systems showed less intense correlations between the linker and TM domains (between 0.1 and 0.5 lower values) and weaker anticorrelations with the ECD of NKG2A (up to 0.4 higher in **CHOL**). In the negative **POPA**, the ECD of NKG2A showed low correlations with TM and linkers of NKG2A (0.1) and even had no anticorrelations with the ICD of NKG2A. Overall, these results suggest that communication between domains of the NKG2A/CD94 protein complex was affected in all membrane models with less intense correlations and anticorrelations compared to when the complex was inserted in a standard **POPC** membrane.

Principal component analysis (PCA) was performed to assess and identify the large-scale collective movements of the receptor (Figure S6). As simulation times that could comprehensively describe large-scale movements of such macromolecular systems are out of reach for the current computational capabilities, this method of essential dynamics can effectively be applied to provide the first clues. From the scatter plots, a difference could be seen in the size of the 2D plot, with the negative **POPA** membrane system having a smaller scatterplot and the **DLPC** and **CHOL** systems having a larger scatterplot. The cumulative contribution to the total variance showed that the first two principal components accounted for at least 60% of the total variance with the figure exceeding 80% for the **DLPC** system. The high values in these systems are a consequence of the receptor being embedded in the membrane, which restricts its movement.

The essential dynamics of the system, obtained by projecting the first principal component (PCA 1) onto the averaged structures of the complex (Figure S6c), revealed a rotational effect on the ECD of NKG2A/CD94/HLA-E in all systems. The direction of eigenvectors was more horizontal in CD94 when the standard **POPC** was used^17^ and with the direction being more vertical in the negatively charged **POPA** membrane. In the mixed **CHOL** system, some eigenvectors of the NKG2A protein pointed downward. In the TM region of the **DLPC** system, the eigenvectors were parallel to the α-helices of the membrane and pointed toward the linkers. Since the α-helices in the membrane are more inclined so as not to protrude from it, the dynamics of the TM region changed accordingly, which may also affect the IC and linker regions, possibly resulting in a more horizontal rotation of the receptor head and an upward movement of the IC region. A smaller but similar effect is observed with **CHOL**, which also exhibits a small degree of horizontal movement in the CD94 transmembrane domain.

Overall, global changes in the dynamics of the NKG2A/CD94/HLA-E complex, coupled with visual observations, indicate sizable effects of the membrane composition on its fluctuations and dynamics, as well as receptor domain communication. Following this initial assessment, we specifically analyzed the intracellular (IC/ICD), transmembrane (TM/TMD), and extracellular (EC/ECD) domains to elucidate more specific effects of different membranes on the receptor structure.

### Interactions with Negatively Charged Phospholipids and Membrane Curvature Control the Intracellular ITIM Exposure

To begin, we investigated changes in the IC region of the receptor to determine whether the membrane structure can influence the behavior of the critical ITIM tyrosine residues responsible for signal transduction by the receptor. The distances between the oxygen atoms of the side chains of Tyr8 and Tyr40 were calculated and its time dependence was plotted during the simulation (Figure S7). The average distances were between 10 and 20 Å for all protein–membrane models. The lowest values were observed for the gel-like **DPPC** membrane (12.5 ± 0.9 Å), followed by the negative **POPA** (13.8 ± 0.6 Å) composition. The saturated **DLPC** (16.8 ± 1.1 Å) and standard **POPC** (18.2 ± 1.2 Å)^17^ membranes exhibited moderate fluctuations at higher distance values, while the mixed **CHOL** (18.9 ± 4.3 Å) had a large peak during its trajectory but otherwise had a lower distance than **POPC** for most of the simulation run.

**Figure 5:**
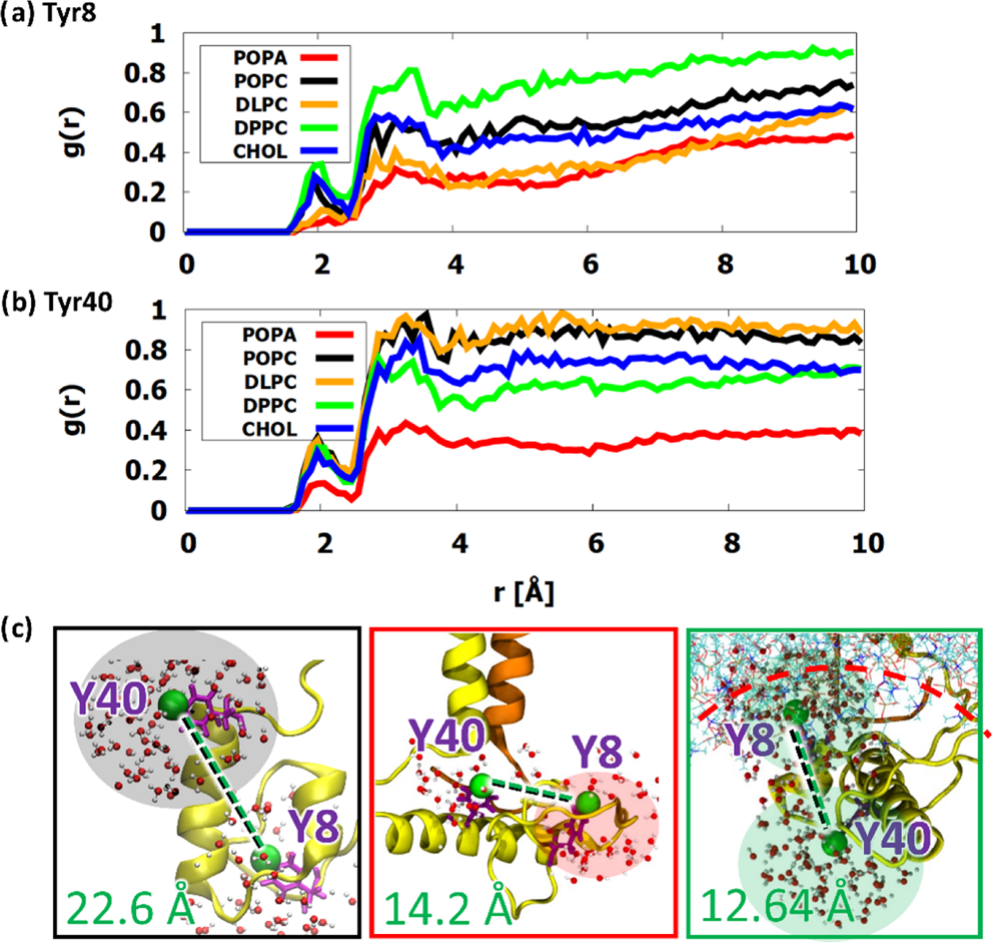
Radial distribution functions (RDF) of water molecules around the ITIM tyrosine oxygen (OH) atoms of (a) Tyr8 and (b) Tyr40. Images under panel (c) show the distribution of water molecules around both ITIM regions and the average distances between tyrosine OH atoms for three of the membrane systems: **POPC** (left),^17^**POPA** (middle), and **DPPC** (right). In the **DPPC** system, the curvature of the membrane lipids is highlighted in red.

Next, we analyzed the radial distribution of water molecules around the ITIM tyrosine oxygen atoms in radial distribution functions (RDF) (Figure 5). A clear trend can be observed, with the negative **POPA** system having the lowest RDF values, suggesting that in this membrane composition it is the least exposed to water molecules, and therefore protein kinases are likely to be largely prevented from effectively accessing its ITIM regions. In other protein–membrane systems, the values were dependent on the specific tyrosine residue—standard **POPC** showed good exposure to water molecules at both residues,^17^ while the saturated **DPPC** and **DLPC** membranes showed good exposure at only one of their ITIMs. Overall, the effect of increased membrane curvature and protrusion angle had a moderate effect on the IC behavior.

Visual inspection of **DPPC** revealed that the slight inward curvature of the membrane in the IC region likely affects the positioning of the IC domain of the NKG2A protein (Figure 2). This would explain the small distance between the ITIMs and yet some of the highest RDF values, which are due to the higher water accessibility between the protein and the membrane lipids. As expected, NKG2A was glued to the surface of the membrane by **POPA**.

To highlight these physical differences, we measured the distance between the centers of mass of the first 45 residues of the NKG2A protein and the transmembrane domain (TMD) (Figure S8) and found that the negatively charged **POPA** was a clear outlier, as its distance was more than 10 Å less than what we observed in all other models. The saturated short **DLPC** membrane had the highest distance of 52 Å throughout the simulation, which can be attributed to the different protrusion angle of the protein with respect to the lipid bilayer. Contact analysis revealed that **POPA** formed by far the most contacts with membrane lipids (Figure S9). **DLPC** and **DPPC** also formed transient interactions in certain parts of the trajectory, while NKG2A residues 1–45 of **POPC**(17) and **CHOL** were rarely detected in close proximity to the membrane, suggesting that more loose conformations that may be more exposed are prevalent.

Finally, we wanted to examine if the membrane-proximal part of NKG2A and the first three residues of CD94 came into close proximity, as it likely correlates with the ability of the two proteins to interact in the ICD (Figure S10). Here, it was the saturated **DPPC** in which stable interactions between NKG2A and CD94 appeared to form as the ability to separate due to membrane curvature decreased. **POPC** had the second lowest separation,^17^ which may be a factor in its ability to expose its ITIM regions better than other models. This is also partially confirmed by secondary structure analysis (Figure S11), which shows that the **POPC** and **CHOL** systems had the largest reduction in the IC α-helical size, while the **POPA** membrane enabled the formation of more helical structures in the region around residue 60. The importance of secondary structure formation has been highlighted as part of the mechanism by which ITIMs are less exposed and therefore less likely to undergo phosphorylation.^10^

### Changes in the Membrane Thickness Drastically Distort the Angle between the Transmembrane Helices and the Lipid Bilayer

**Figure 6:**
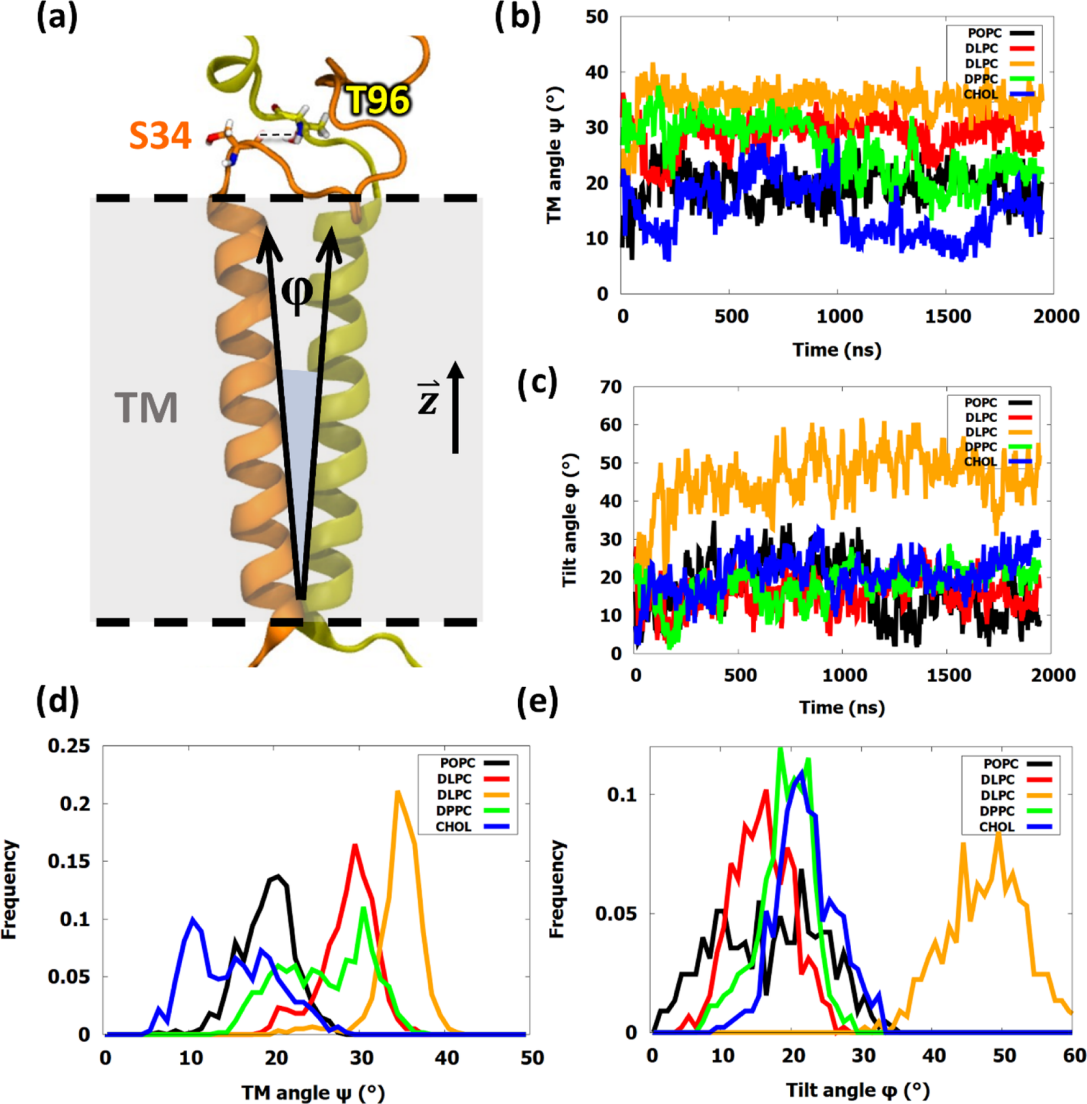
Angle between transmembrane helices of NKG2A and CD94 proteins. (a) Angle φ is showcased between two vectors, corresponding to the top and bottom of each helix. At the top, a plausible hydrogen bond, Thr96^NKG2A^–Ser34^CD94^, is shown. The TM angle ψ is defined as the angle between the NKG2A helix and vertical vector . (b) TM angle ψ as a function of simulation time and (c) tilt angle as a function of time. (d) Frequency distribution of TM angles ψ, expressed as a percentage of all snapshots. 1° intervals were chosen in data processing. (e) Frequency distribution of tilt angles φ, expressed as a percentage of all snapshots. 1° intervals were chosen in data processing.

The transmembrane regions of the vast majority of the signaling proteins play a crucial role in the transmission of a signal across a membrane.^49^ Measurement of the angle between the two α-helices revealed significant differences among the five simulated models (Figure 6). The short saturated **DLPC** membrane especially stood out here due to the oblique positioning of the TM regions of NKG2A and CD94 proteins. This was reflected in the angle between the helices, reaching 35°. The standard **POPC**(17) and mixed **CHOL** systems showed the lowest values, which could be a sign of increased signaling ability, as the negative **POPA** membrane again showed higher angle values by about 10°.

By measuring the angle between the vector formed by residues Val73 and Val92 of NKG2A and the vertical axis vector, we then investigated the degree of tilting of the protein complex relative to that of the membrane (Figure 6). Unsurprisingly, the saturated short **DLPC** system maintained the highest angles, averaging a 45° tilt. Other models were overall quite similar, though the standard **POPC** displayed slightly lower angles in the latter half of the trajectory, reaching almost a perfect vertical orientation at 0°.^17^

This observed difference is probably associated with the positioning of the linker regions in both NKG2A and CD94, which are proximal to the membrane lipids and can form both transient and permanent interactions not only with the membrane but also via protein–protein interactions between the linker regions themselves. By comparing the linker–linker contacts (Figure S12) and the linker–membrane contacts (Figure S13), it is clear that the negative **POPA** and mixed **CHOL** form the majority of contacts in both cases. The two saturated **DLPC** and **DPPC** systems formed a low number of linker–membrane contacts, while the number of linker–linker contacts was comparatively higher for the standard **POPC**.^17^ Interestingly, the specific hydrogen bonds OG@Ser34^CD94^ and OG@Thr96^NKG2A^, which were assumed to have an effect on the correct positioning of the α-helices,^17^ were only formed in the **POPC**(17) and **DLPC** systems (Figure S14), although in the mixed **CHOL** model several other hydrogen bonds were formed, resulting in a surprisingly elongated and parallel linker shape that may still allow specific α-helix positioning in the TMD. This result highlights the complexity of signaling events, indicating that more studies may be needed to fully elucidate the exact signaling mechanism, particularly in the case of cholesterol-rich membranes.

### Extracellular Region Is Only Moderately Affected by the Change in the Membrane Composition

One of the major clusters in the standard **POPC** signified a tilt of the NKG2A/CD94 receptor head, indicating a high flexibility of the receptor structure.^17^ Otherwise, the ECD domains in the other simulated models remained upright with minimal interactions with the membrane lipids in all models (Figure 2). This is further evident from the contact analysis of the receptor head and membrane lipids (Figure S15), which shows that the saturated **DLPC** and **DPPC** systems formed transient interactions in small parts of the trajectory. Interestingly, **POPC** formed the most interactions, resulting from its higher flexibility in the ECD.^17^

**Figure 7:**
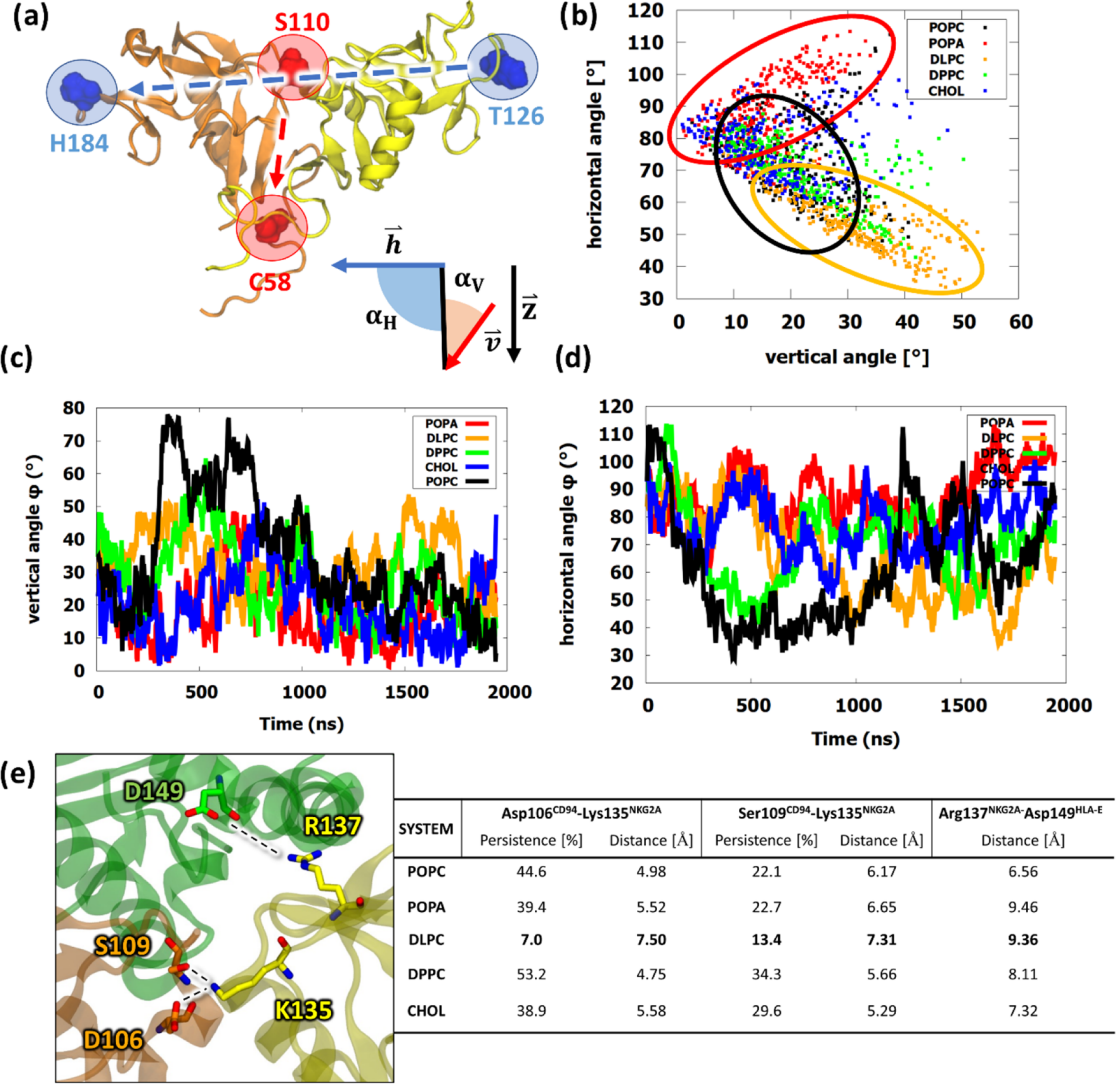
Bending angle analysis of the extracellular NKG2A/CD94 receptor domain. Angles were calculated with respect to the vertical unit cell vector . The histogram box size for frequency distributions is 3°. (a) Visual representation of the selected vectors. The vertical vector is shown in red (), while the horizontal vector is colored in blue (). The schematic shows the horizontal (α_H_) and vertical (α_V_) angles.^17^ (b) 2D angle plot, showing the conformational space of the receptor head with vertical angles on the *x*-axis and horizontal angles on the *y*-axis. The colored circles represent the biggest differences in the conformational space between models. (c) Vertical angle frequency distribution using a vector between the centers of mass of Cys58–Ser110. (d) Horizontal angle frequency distribution using vector His184–Thr126. (e) Summary of the average distance and persistence of bonds Asp106^CD9^–Lys135^NKG2A^ and Ser109^CD94^–Lys135^NKG2A^, as well as the distance between Arg137^NKG2A^ and Asp149^HLA-E^. Persistence indicates the percentage of frames in which contact is established between residues within a threshold of 4 Å.

To evaluate the specific inclination of the receptor head, we calculated horizontal and vertical angle frequency distributions and time dependences corresponding to the two possible inclinations of the ECD (Figures 7, S16, and S17). The vertical angles generally did not exceed 50° in the latter part of the trajectory, suggesting that the membrane composition does not affect the dynamics of the ECD to the same extent as the absence of the HLA-E ligand.^17^ Nevertheless, we could see differences in the 2D angle plot, where the short-lipid **DLPC** membrane points were more scattered, while the negatively charged **POPA** points were grouped in a smaller area. The vertical angles of other models were more consistent and closer to 0°, and the horizontal angles between models were more spread, with **DLPC** deviating toward 50° and **POPA** toward 100°. Here, we can also notice that **DLPC** had many conformations of the ECD that had low vertical and horizontal angles at the same time, setting it as a clear outlier in both frequency plots. It is likely that the angle at which the protein protruded from the membrane also altered its ECD positioning, highlighting the apparent disruption that an exaggeratedly small bilayer may cause. We also note that while **POPC** interacted the most with the membrane, these interactions did not affect the core dynamics and functionality of the receptor head very much, showing only in slightly higher vertical angles and no difference in the horizontal positioning.^17^

Within the receptor head, we finally analyzed the hydrogen bonds between Asp106^CD94^–Lys135^NKG2A^ and Ser109^CD94^–Lys135^NKG2A^, which have been identified as potentially significant for signal transduction (Figures 7e, S18, and S19).^50^ Interestingly, both hydrogen bonds were significantly less likely to form only in the **DLPC** bilayer, also corresponding to a high distance between Arg137^NKG2A^ and Asp149^HLA-E^. **POPA** and other models showed moderately high persistence values for these bindings, suggesting that the effect of membrane composition may be less significant for ECD due to HLA-E binding, which is likely to have a stronger effect in this case. However, the hydrogen bond network may possibly still be impacted by drastic changes to the TMD, highlighting the complexity of transmembrane signaling.

## Discussion

In the past, the dynamics and function of transmembrane proteins have been challenging to study.^51^ In part, this is also due to the staggering complexity and variation of lipid structures composing the lipid bilayers of cells, leading to subtle differences that can have a major influence on the final physiological outcome. Many of these effects have been difficult to study computationally as large membrane systems must also be accompanied by long simulations to ensure adequate equilibrium and coverage of a sufficiently large conformational space to afford relevant results.^52,53^ Recently, a substantial increase in the available computational power and development of new techniques that allow for an efficient study of larger macromolecular systems over longer periods of time using molecular dynamics simulations has been made.^54^ The second important aspect was the improvement of the accuracy of protein structure determination with methods like cryo-EM^55^ and superior 3D structure prediction by AI-based methods such as Alphafold 2.^21,22^ Both factors allow for a more accurate assessment of transmembrane proteins, such as in the case of the NKG2A/CD94 receptor.

In this study, we utilized our generated 3D structure of the NKG2A/CD94/HLA-E model.^17^ From a combination of the crystal structure of the ECD (PDB ID: 3CDG)^20^ and Alphafold 2 predictions of the ICD and TMD regions, the mechanistic details of signal transduction by the receptor have previously been investigated.^17^ Here, we have focused on an often-neglected component of these complex systems and comprehensively explored the effects of the composition of the membrane on the transmembrane protein dynamics and tried to draw conclusions on the mechanism of signal transduction. To this end, we constructed 5 immune NKG2A/CD94/HLA-E complex–membrane systems each with somewhat different properties of the phospholipids that compose the generated membrane: a standard **POPC**,^17^ negatively charged **POPA**, saturated (short-lipids) **DLPC**, saturated **DPPC**, and finally an even more advanced lipid/cholesterol **CHOL** mixed model. We would like to emphasize that these membrane models were not chosen for their biological significance as cell membranes are diverse in their lipid composition but to represent more extreme changes in the possible lipid environments and more efficiently study their effects on the protein complex, assessed by 2 μs molecular dynamics simulations. Analysis of membrane properties, such as area per lipid values, indicated that the membranes were well-equilibrated. Based on the observed trends in the conducted simulations, we found that overall the ICD and TMD of NKG2A/CD94 were significantly affected by the change in the membrane composition, while the dynamics of the receptor head with the bound HLA-E ligand (i.e., EDC) was affected to a lesser degree. In this portion, the formed protein–protein interactions with HLA-E dominate over lipid contacts because a larger binding area precludes upright ligand binding, avoiding extreme deviations from a 90° vertical angle. Small differences were noted in the strength of correlations in the linker and TM regions, together with global changes in mobility, with systems like the standard **POPC** and mixed **CHOL** displaying higher flexibility. The crossing angle of the two α-helixes in the TMD was also indicative of the membranes’ effect on receptor structural changes.

Past studies indicate that a negatively charged membrane surface has a profound effect on signal transduction of transmembrane proteins as the negative surface potential attracts positively charged molecules from the cytoplasm, with anionic phospholipids also being able to assemble into nanoclusters and affecting signaling pathways.^56^ In the case of the NKG2A component, such a membrane likely protects the tyrosine residues (Tyr8 and Tyr40) in the ITIM regions from phosphorylation by shielding and sequestering them into the bilayer in the absence of HLA-E, caused in part by electrostatic interactions with basic protein residues.^57^ The high concentration of anionic lipids in **POPA** increased the strength of these interactions, sticking the otherwise mobile ICD of NKG2A/CD94 to the membrane via salt bridges with Arg and Lys residues. We therefore suggest that a highly increased presence of **POPA** and other negatively charged lipids in the membrane may hinder signal transduction at the local level. In the ECD, this effect was less pronounced as the protein did not differ in the amount of interaction with the membrane, likely because the interactions with HLA-E were dominant.

The thickness of the membrane has a clear influence on the positioning of the TMD. Generally, membrane proteins perturb the surrounding bilayer through deformations when undergoing conformational changes, necessitating that the protein and bilayer are well adapted to each other for optimal energetics.^58^ In the case of the α-helix dimers, biological membranes can adapt to structural deformations since despite poor hydrophobic match, mechanisms such as dimer tilting or local thickness perturbations are able to preserve the stability of α-helices.^59^ The **DLPC** membrane contains lipids with 12 saturated carbon atoms, which are 4–6 carbon atoms shorter than the typical standard length of a **POPC** membrane. This results in a predictable decrease in membrane thickness from 37 to 30 Å, which has a significant effect on the positioning of the NKG2A/CD94 TMD.^60^ In order to accommodate all of the hydrophobic transmembrane α-helices, these helices tilt extremely parallel to the membrane axis. This also changes the positioning of the helices relative to each other, possibly affecting signal transduction.^17^ The ICD exposure of the ITIMs in **DLPC** varied, with one tyrosine being highly exposed and the other being more shielded. The ECD also interacted slightly more strongly with the membrane lipids and the receptor head taking different conformations compared to other models. Overall, the lipid length has a moderate effect on signal transduction of **DLPC** as it influences the angle of protrusion of the linker regions from the membrane. Our simulations have discovered that the primary mechanism for resolving hydrophobic mismatch was helix rotation; however, *in vivo* local membrane changes might play a more substantial role.^61^

The degree of lipid saturation can also influence cell signaling events through properties such as elasticity and stiffness of the membrane.^11^ The presence of saturated lipids may also alter the critical temperature of the membrane, which alters the protein membrane activity.^62^ We visually observed a curvature of the **DPPC** membrane during the simulation, likely an effect of its high transition temperature of 41.3 °C.^63^ This resulted in a gel-like state of the membrane that was also observed in other studies and resulted in a curvature of the membrane to a tilted or cross-tilted phase.^43,46^ Specifically, our system visually resembles a disordered gel phase, which another study has also found through molecular dynamics simulations.^44^ Interestingly, this change in the membrane also affected the intracellular region of NKG2A as its dynamics around a curved membrane was different, resulting in greater exposure of ITIMs and thus potentially easier access to Src kinases for subsequent phosphorylation. The high number of NKG2A-CD94 contacts near the TM region and relatively high amount of contacts with the membrane also resulted from this effect. This confirms that the phase behavior of biological membranes and ordered domains may also influence the workings of membrane-embedded proteins.

**Figure 8:**
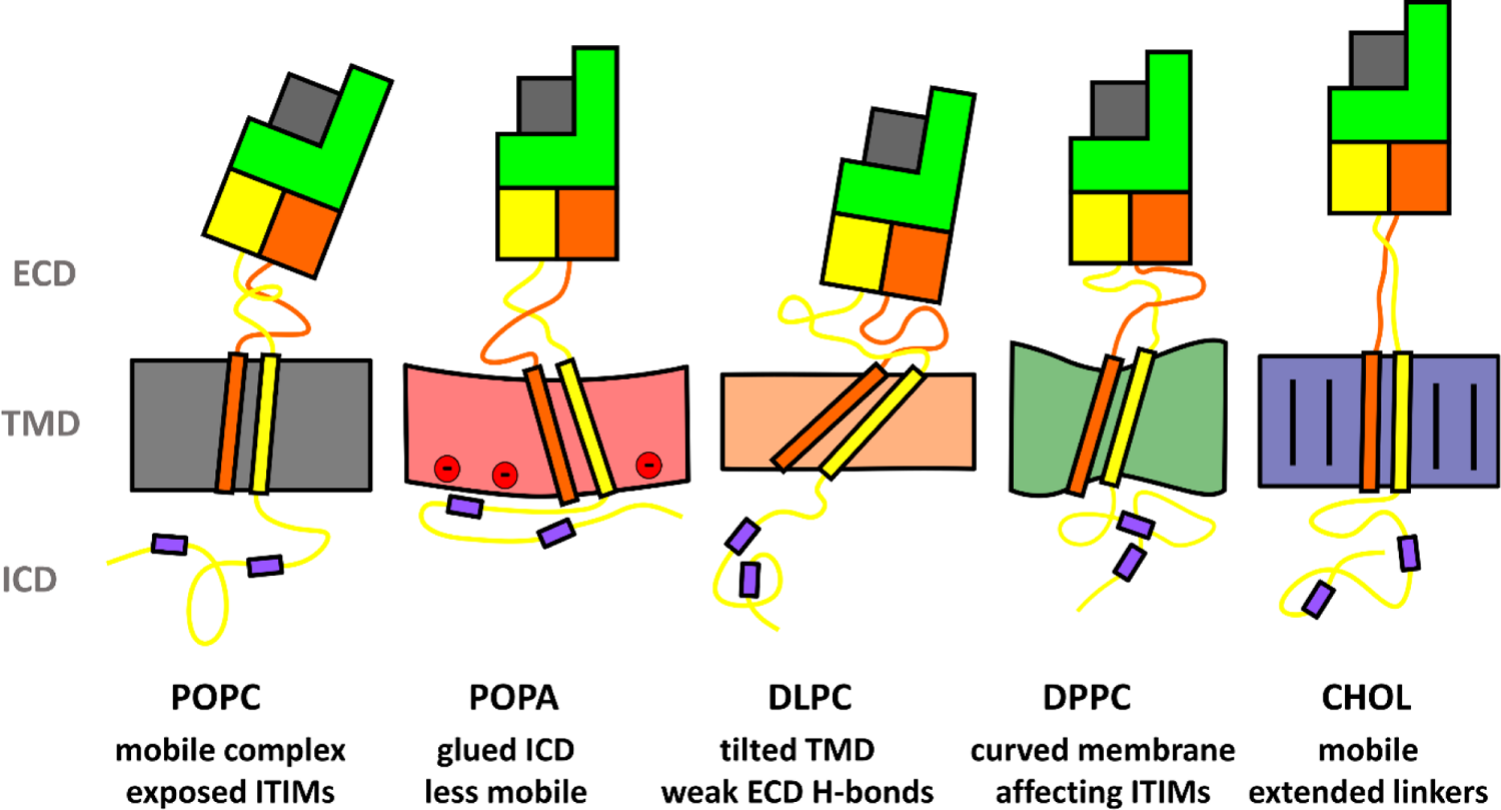
Schematic representations of the key changes observed under different membrane compositions. NKG2A is shown in yellow, CD94 in orange, and HLA-E in green. The NKG2A ITIM regions are represented as purple rectangles. (**1**) In the standard **POPC** membrane, the ECD is mobile and the ICD ITIM regions are exposed to water molecules. (**2**) In **POPA**, the negative membrane lipids strongly interact with NKG2A ICD, gluing the intracellular region to the membrane with a small curvature that causes a moderate TMD tilt. (**3**) Thin saturated **DLPC** membrane forces a significant tilt of the TMD helices. The hydrogen bond network within the ECD is weakened. (**4**) Saturated **DPPC** membrane is in a gel-like state, inducing significant curvature, which influences the positioning of the ICD. (5) Mixed lipid/cholesterol **CHOL** displayed extended linkers; otherwise, no major changes were observed.

Cholesterol is known to have a significant effect on immunoreceptor signaling.^64^ Our simulations were unable to assess and detect the organization of cholesterol molecules into “lipid raft” domains in the **CHOL** model, which may be responsible for the differences in signaling through activating and inhibitory receptors.^65^ Experimentally, lipid rafts were found to be excluded from the site of NKG2A/CD94 contact with the ligand, which may also impede activation signals.^66^ At the local protein level, the main difference in our simulation using the **CHOL** setup was in the organization of the linker domains and the distance of the ECD from the membrane, as evidenced by lower correlations to this region, although the TMD and IC regions were minimally affected and showed little difference in the dominant movements of the TMD. These observations could imply that cholesterol may instead have a substantial impact on the membrane organization and receptor clustering on a larger scale for immune receptors such as NKG2A/CD94, even though some other proteins can interact with it directly.^67^ General membrane parameters such as an increase of lipid order parameter values and decrease of area per lipid were comparable to the values in other works and could play a role in shaping interactions between NKG2A and lipid molecules.^68^ To briefly summarize the observed effects of different membrane compositions on the dynamics and signal transduction of the NKG2A/CD94 protein as observed in our simulations, we have created Figure 8, which schematically presents the key findings.

To further validate the conclusions of the simulations, we simulated two additional replicas for each system in length of approximately 1 μs. The underlying conclusions have been confirmed by the extended set of simulations, although some deviations were observed that should be highlighted (Figures S20–S27). The membrane parameters across all replicas were incredibly consistent, indicating successful equilibration and stability (Figure S20). The **DPPC** curvature due to the gel state of the membrane was observed in all replicas and likely had an effect on the positioning of the ICD (Figure S21). RMSF values indicate that one of the **POPA** replicas exhibited higher flexibility values, but the average flexibility of **POPA** still remained relatively low and all systems were slightly closer in flexibility when considering all replicas (Figure S22). Additionally, all **POPA** replicas formed high amounts of interactions with membrane lipids (Figure S23). The exposure of ITIM tyrosine residues to water had among the highest fluctuations between some systems, but the averaged calculations confirm our observations with **POPA** tyrosines displaying the lowest exposure to waters and **POPC** and **DPPC** among the highest (Figure S24). Angles in the TMD and ECD were in very good agreement across the replicas, particularly in **DLPC** that displayed the highest TMD angles (Figures S25 and S26). However, H-bonds in the ECD of **DLPC** were not weakened to the same degree, which affects the confidence of this particular conclusion and suggests potentially a more complex molecular recognition occurring in this system (Figure S27).

While this study reveals valuable atomistic insight into the key effect of the membrane composition on protein dynamics, certain limitations have to be noted. Because only the extracellular structure of the simulated NKG2A/CD94 protein has been experimentally determined to date, the transmembrane and intracellular domains were modeled and thus lack structural validation. These simulations also highlight a certain amount of variability that can occur even between replicas of the same system, warranting careful interpretation of the data. Finally, as the membrane conditions are exaggerated and limited in their number, they do not represent accurate biological membranes and should not be interpreted as such. Since cell membranes are a complex environment, the construction of larger systems with multiple lipid species, simulated at longer time intervals, would be required to give a much more comprehensive and detailed picture of immune cell signaling.

Taken together, we have shown that the effect of membrane composition is not to be neglected when investigating the dynamics and mechanism of transmembrane proteins. Our study represents another much-needed large systematic computational evaluation of the membrane parameter’s effects on the receptor signaling process, taking the highly biologically relevant NKG2A/CD94/HLA-E protein complex as the investigated case. Considering the effects of negative membrane potential, thickness, and the resulting hydrophobic mismatch or saturation and cholesterol levels, protein behavior and subsequently signal transduction are sensitive to changes in membrane composition and should be considered during studies that involve membrane systems, such as those that involve cancer and senescent cells. Additionally, studying the effects of membrane composition using molecular dynamics simulations can provide a more comprehensive picture of the signal transduction process as well as a springboard for future biochemical experiments targeting the change in receptor signaling events.

## Data Availability

All molecular simulations, analysis, and visualization were performed with widely used programs available freely for academic institutions: Gromacs 2019, Amber20 and AmberTools20, VMD 1.9.3, PyMol 2.0, and ChimeraX. All procedures and workflows are described in the Methods section. Structure and parameter files are provided in the Supporting Information. Additional data including input files, final structures, and trajectories are available at Zenodo: 10.5281/zenodo.11580928.

## Supporting Information Available

Structure files of the complex systems and topology files. (PDF)
Movie of the simulated models. (MP4)
