# Supplementary material for: All-Atom Simulations Reveal the Effect of Membrane Composition on the Signaling of the NKG2A/CD94/HLA-E Immune Receptor Complex
Source: J Chem Inf Model. 2024 Dec 2;64(24):9374–87. doi: 10.1021/acs.jcim.4c01357 (PMC11684013; doi:10.1021/acs.jcim.4c01357)
Supplement: Supplementary file 1 — ci4c01357_si_001.pdf [file ci4c01357_si_001.pdf]

SUPPORTING INFORMATION

All-atom simulations reveal the effect of membrane composition on the signaling of the NKG2A/CD94/HLA-E immune receptor complex

Martin Ljubič[†,&], Andrej Perdih[†,&,*], Jure Borišek[†,*]

[†]National Institute of Chemistry, Hajdrihova 19, 1000, Ljubljana, Slovenia

[&]Faculty of Pharmacy, University of Ljubljana, Aškerčeva 7, 1000 Ljubljana Slovenia

Corresponding author*:

E-mail: jure.borisek@ki.si

E-mail: andrej.perdih@ki.si

Table of contents

Supporting figures

Figure S1 3
Figure S2 4
Figure S3 5
Figure S4 6
Figure S5a 7
Figure S5b 8
Figure S6 9
Figure S7 10
Figure S8 11
Figure S9 12
Figure S10 13
Figure S11 14
Figure S12 15
Figure S13 16
Figure S14 17
Figure S15 18
Figure S16 19
Figure S17 20
Figure S18 21
Figure S19 22
Figure S20 23
Figure S21 24
Figure S22 25
Figure S23 26
Figure S24 27
Figure S25 28
Figure S26 29
Figure S27 30

Supporting figures

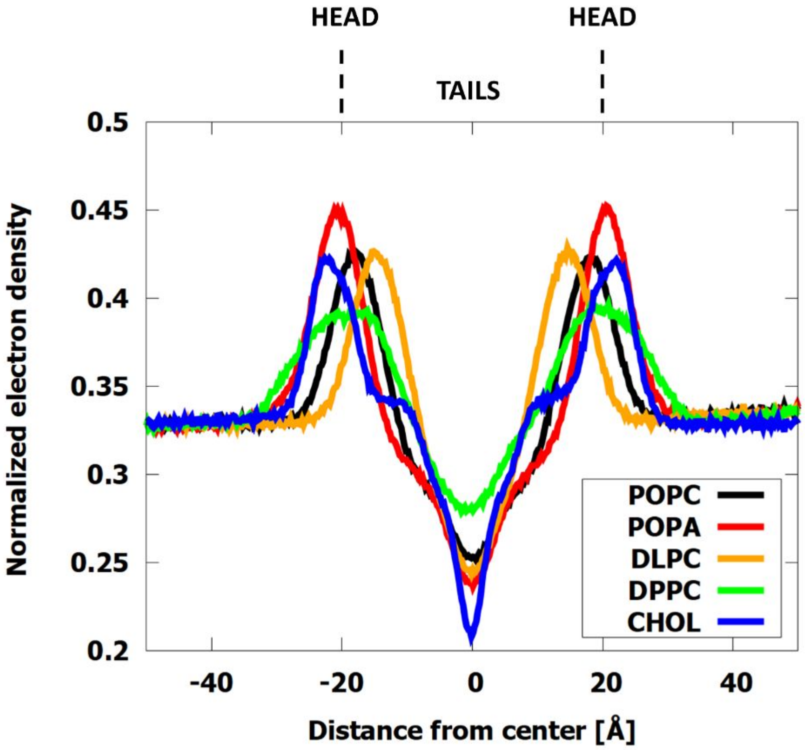

Figure S1. Normalized electron density profiles for the **POPC**, **POPA**, **DLPC**, **DPPC** and **CHOL** models, calculated as a function of the distance from the center of each membrane. Approximate positions of lipid heads and tails are marked above the figure.

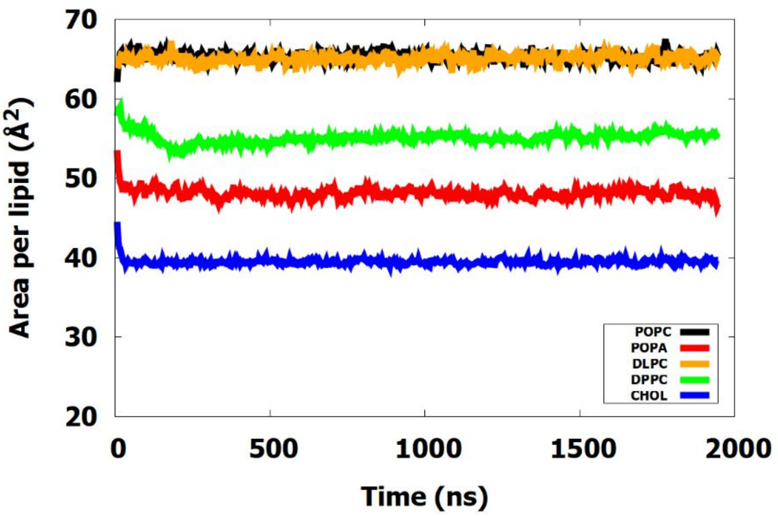

Figure S2. Area per lipid values for the **POPC**, **POPA**, **DLPC**, **DPPC** and **CHOL** models as a function of time during the production run of the simulations.

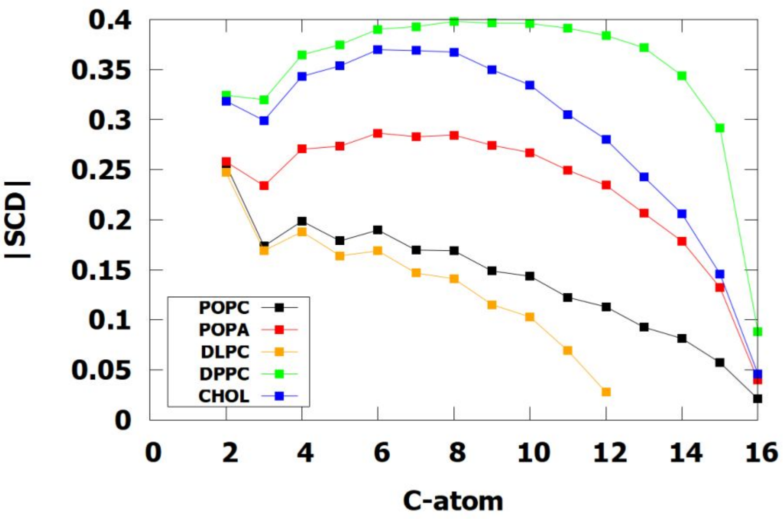

Figure S3. Absolute value of the deuterium lipid order parameters, shown as a function of the C-atom position for the **POPC**, **POPA**, **DLPC**, **DPPC** and **CHOL** models.

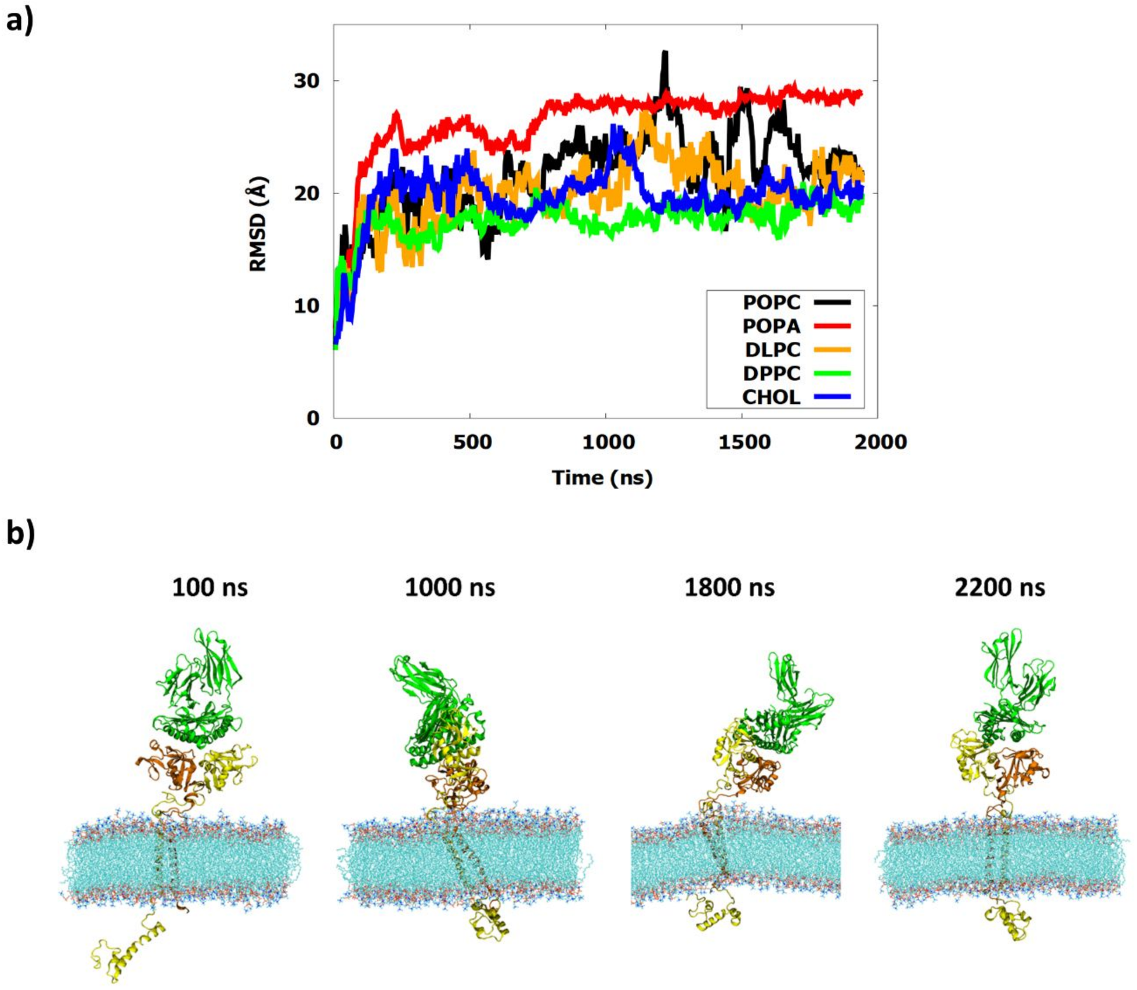

Figure S4. a) Root Mean Square Deviation (RMSD) values for the **POPC**, **POPA**, **DLPC**, **DPPC** and **CHOL** models during the simulation. Only the receptor part of the models was used in the alignment and calculation of the RMSD values. b) Snapshots of the **POPC** system at four different timeframes in the trajectory. Flexibility can be observed particularly in the extra cellular domain (ECD) region of the receptor.

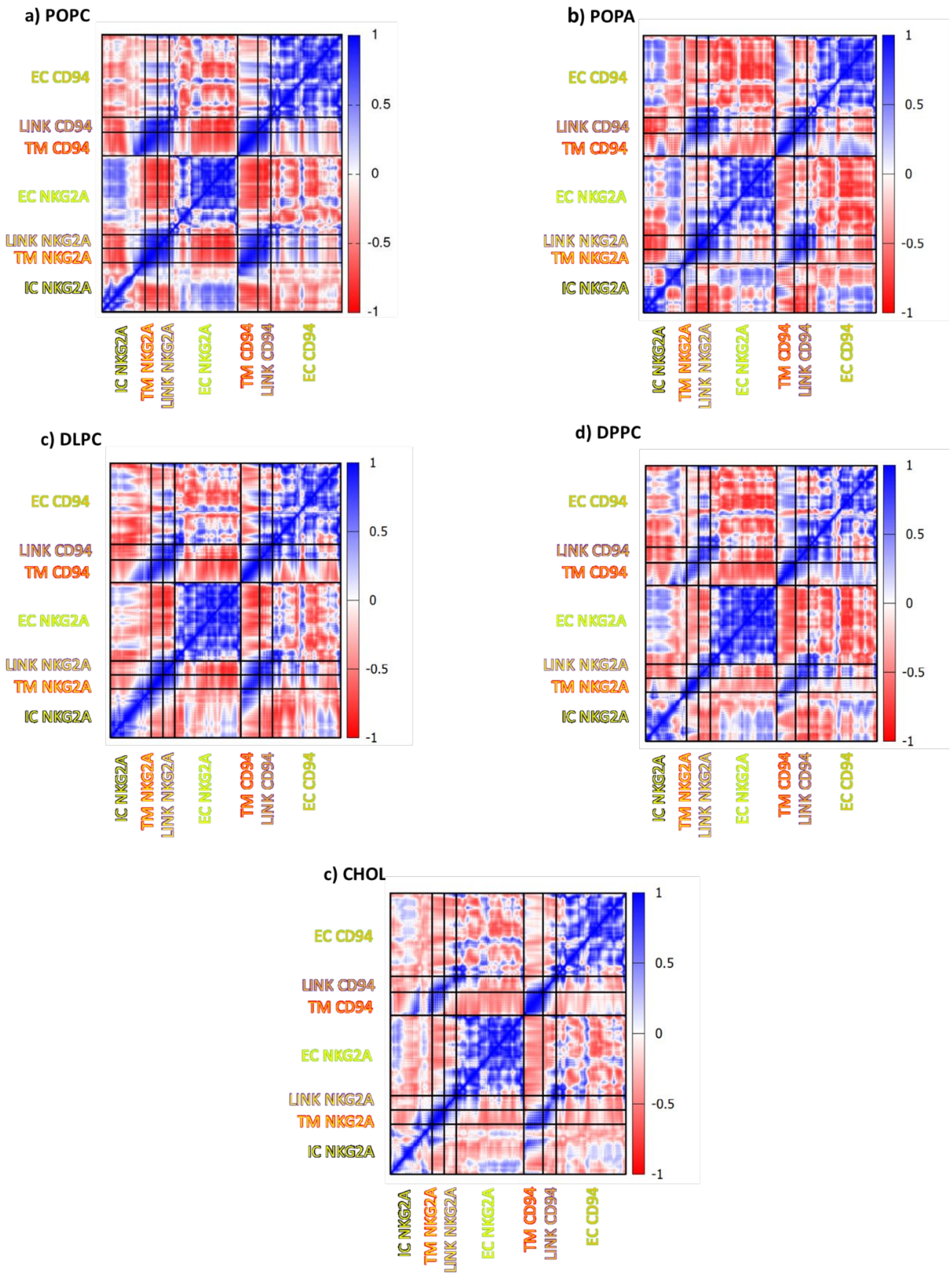

Figure S5a. Non-simplified correlation matrices for the receptor part of the NKG2A/CD94 protein complex of the **POPC**, **POPA**, **DLPC**, **DPPC** and **CHOL** models.

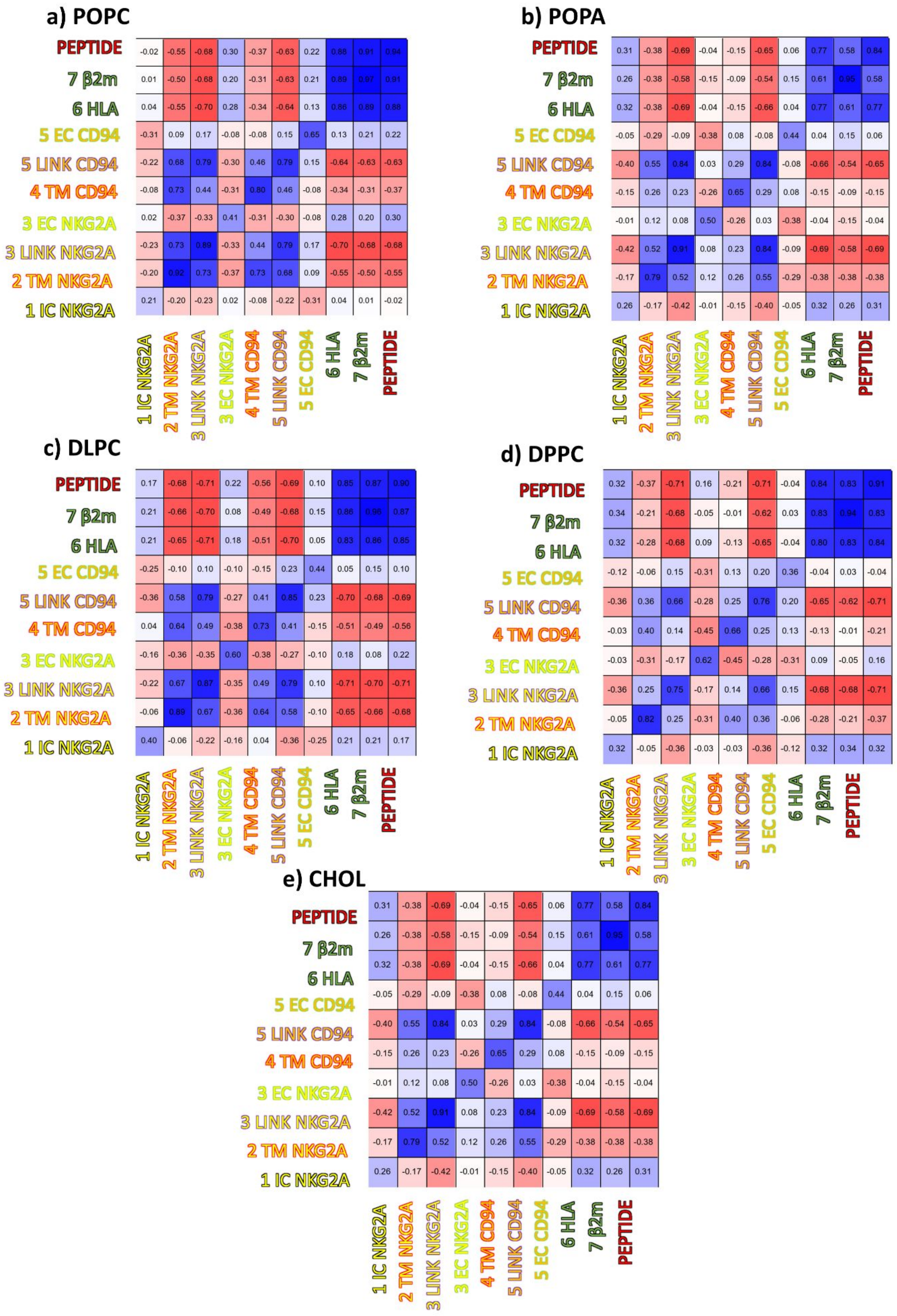

Figure S5b. Simplified correlation matrices with added numerical values for the NKG2A/CD94 protein in the membrane models a) **POPC** b) **POPA**, c) **DLPC**, d) **DPPC** and e) **CHOL**. All frames were aligned to the receptor structure during the correlation matrix calculations.

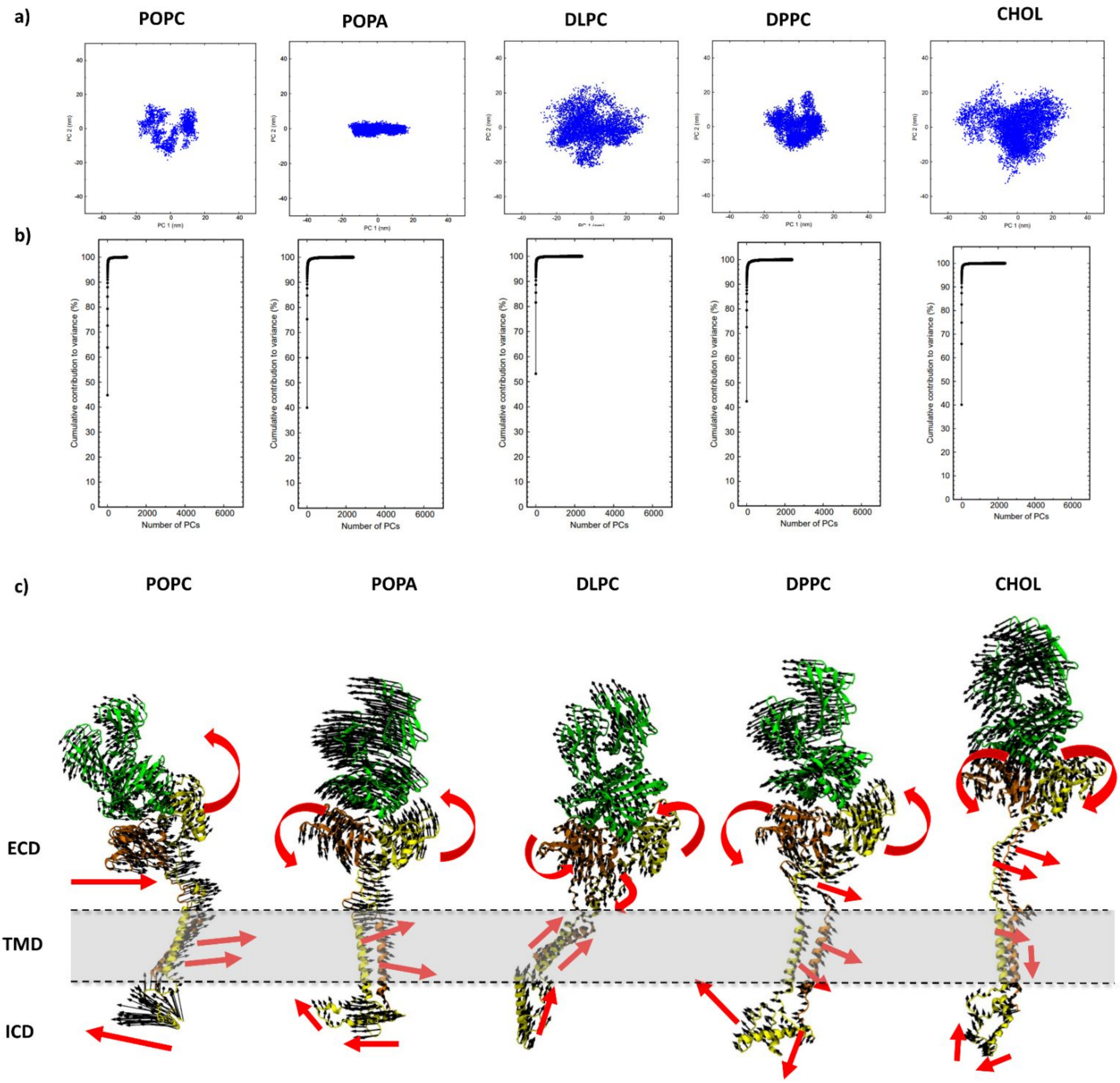

Figure S6. Principal component analysis (PCA) of the NKG2A/CD94/HLA-E protein complex of the **POPC**, **POPA**, **DLPC**, **DPPC** and **CHOL** models as part of the global protein analysis. a) Scatter plots, which represent the projections of the Cα atom displacements along the trajectory onto the first principal component (PC1, x-axis) vs. the projections onto the second principal component (PC2, y-axis). b) Cumulative contribution of the PC's to the overall variance. c) Projections of the first principal mode PC1 onto the 3D protein structures of each of the membrane models. Red arrows have been drawn to help visualize the main motions and the gray area represents the approximate boundary of the membrane.

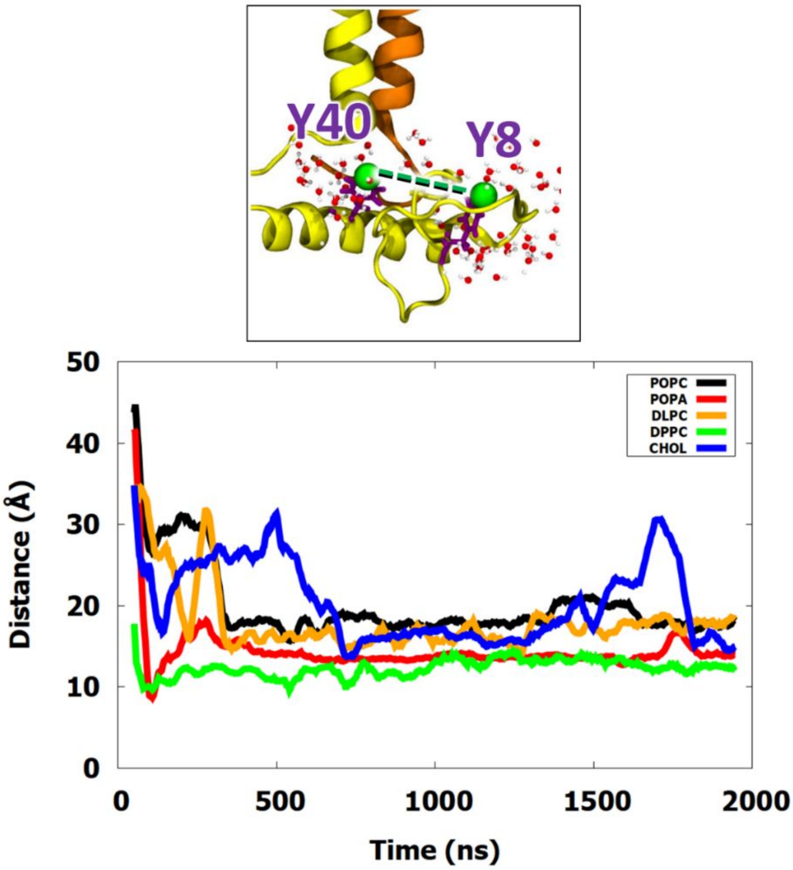

Figure S7. Distance between OH@Tyr8 and OH@Tyr40 of the ITIM regions of NKG2A of the **POPC**, **POPA**, **DLPC**, **DPPC** and **CHOL** models. The oxygen atoms of tyrosine residues are colored green.

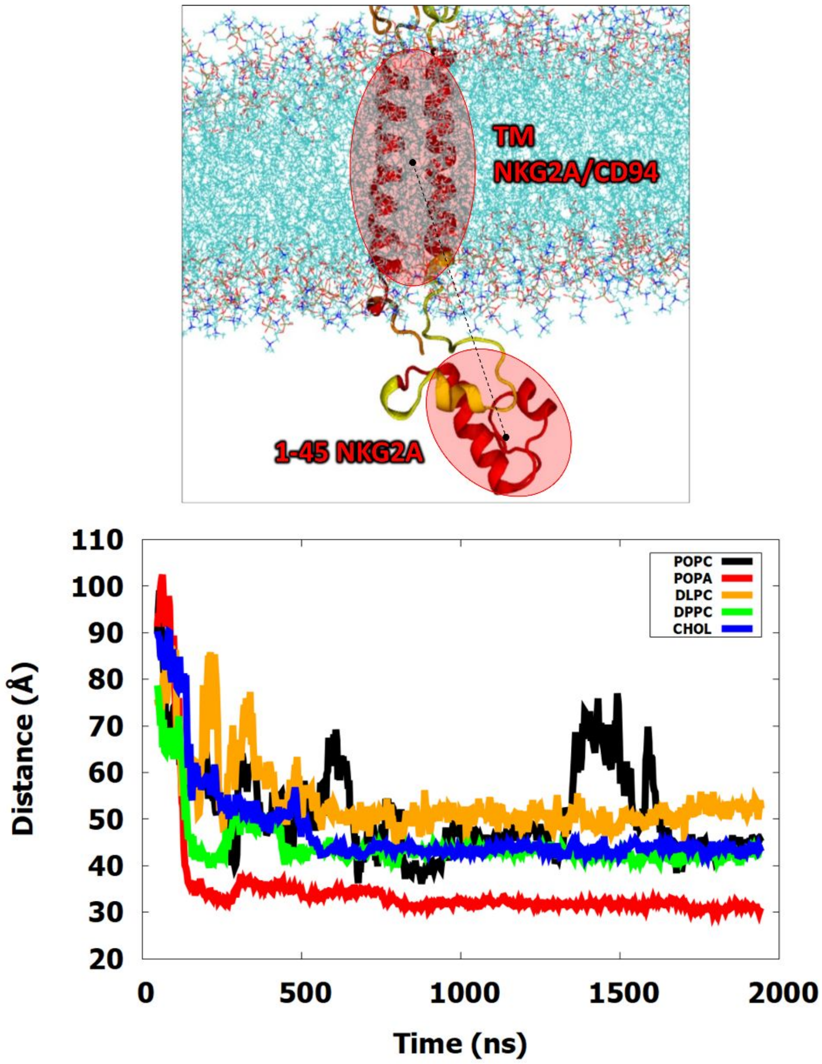

Figure S8. Distances between the center of mass of residues 1-45 of NKG2A and the TM regions of NKG2A/CD94 of the **POPC**, **POPA**, **DLPC**, **DPPC** and **CHOL** models. The two centers of mass are colored in red.

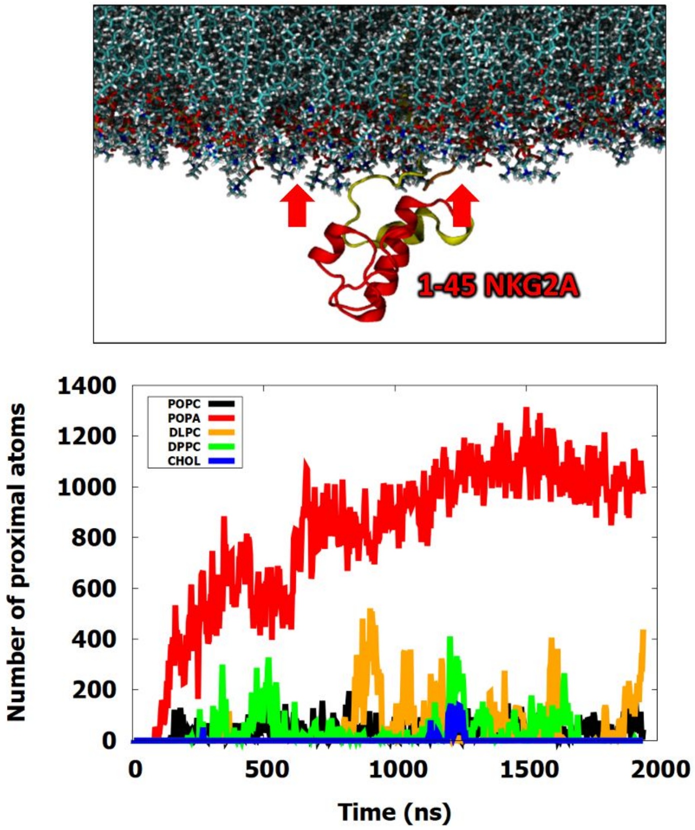

Figure S9. Total number of proximal atomic contact pairs between the IC 1-45 region of NKG2A and membrane lipids of the **POPC**, **POPA**, **DLPC**, **DPPC** and **CHOL** models. The cutoff distance was set to 4 Å. The interacting NKG2A residues are colored red.

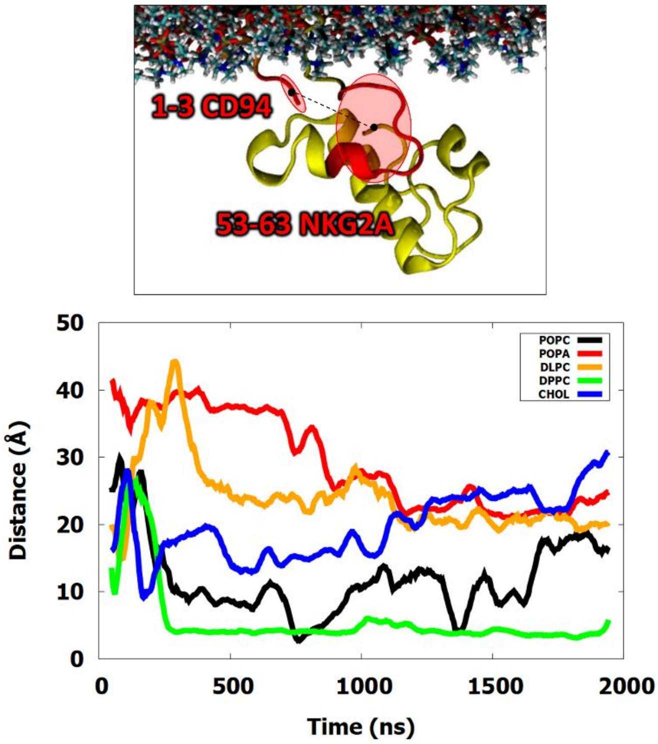

Figure S10. Distances between the center of mass of residues 1-3 of the CD94 protein and residues 53-63 of the NKG2A protein of the **POPC**, **POPA**, **DLPC**, **DPPC** and **CHOL** models. The two centers of mass are colored red.

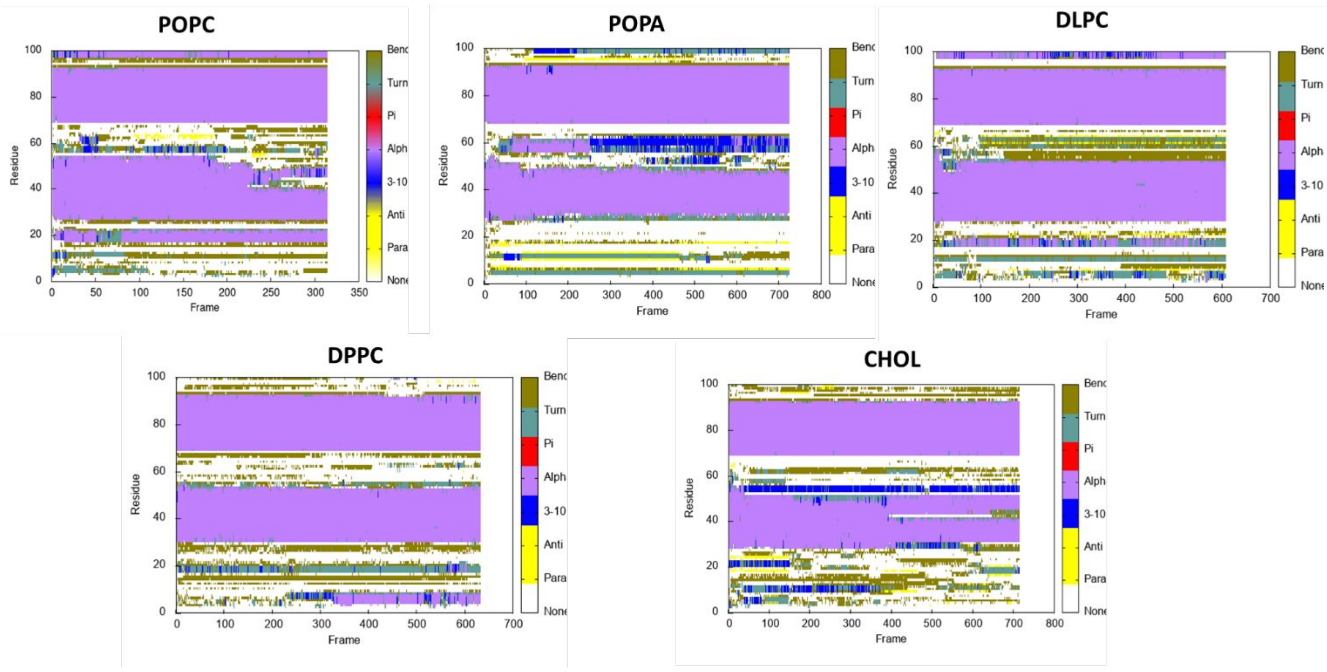

Figure S11. DSSP plots of residues 1 to 100 of NKG2A of the **POPC**, **POPA**, **DLPC**, **DPPC** and **CHOL** models. Colors in each frame represent different types of secondary structure: α-helix (purple), 3-10 helix (blue), π-helix (red), β-sheet (yellow), bend (brown), turn (cyan) and unstructured (white).

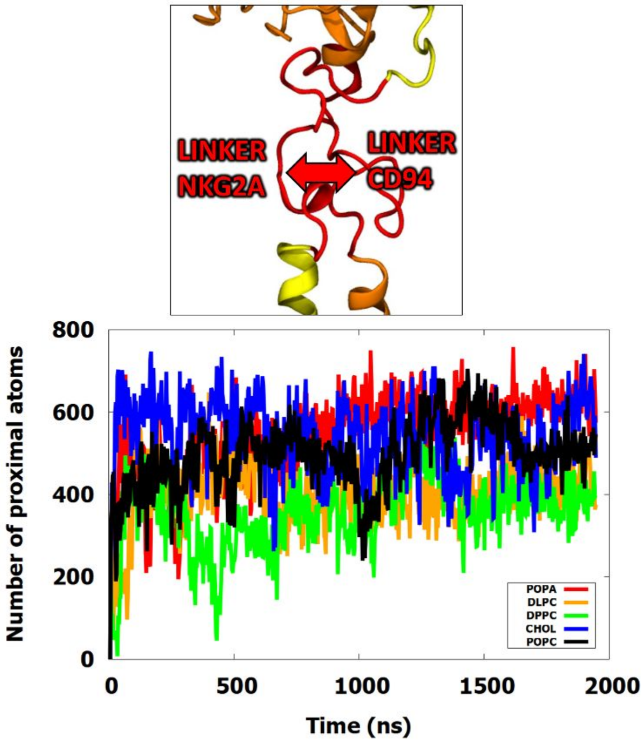

Figure S12. Total number of proximal atomic contact pairs between the linker regions of NKG2A and CD94 of the **POPC**, **POPA**, **DLPC**, **DPPC** and **CHOL** models, as shown in red in the figure. The cutoff distance was set to 4Å. The linker regions are colored red.

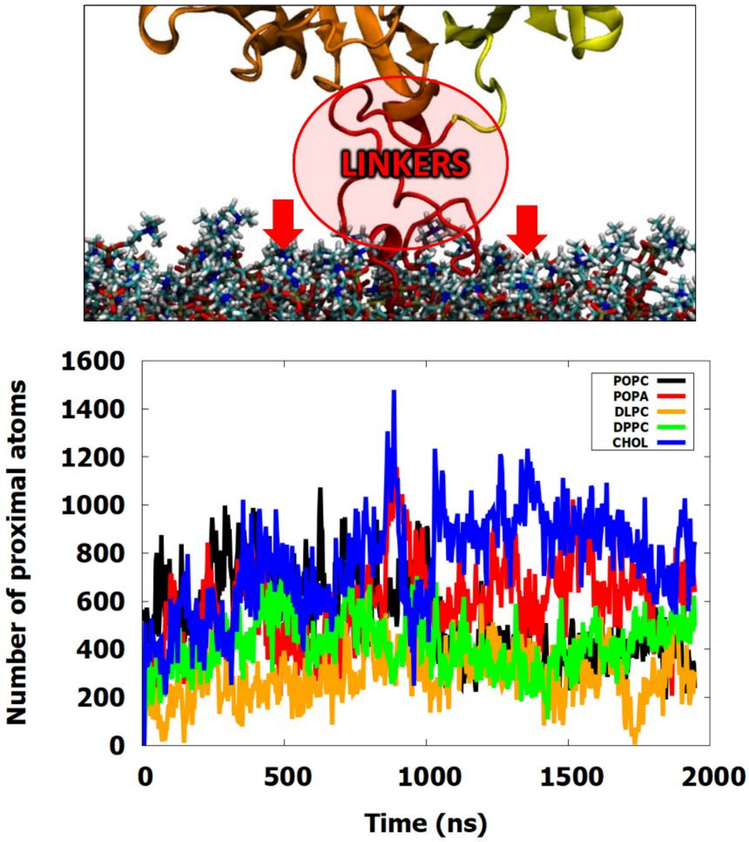

Figure S13. Total number of proximal atomic contact pairs between the membrane lipids and the linker regions of NKG2A and CD94 and membrane lipids of the **POPC**, **POPA**, **DLPC**, **DPPC** and **CHOL** models. The cutoff distance was set to 4Å. The linker regions are colored red.

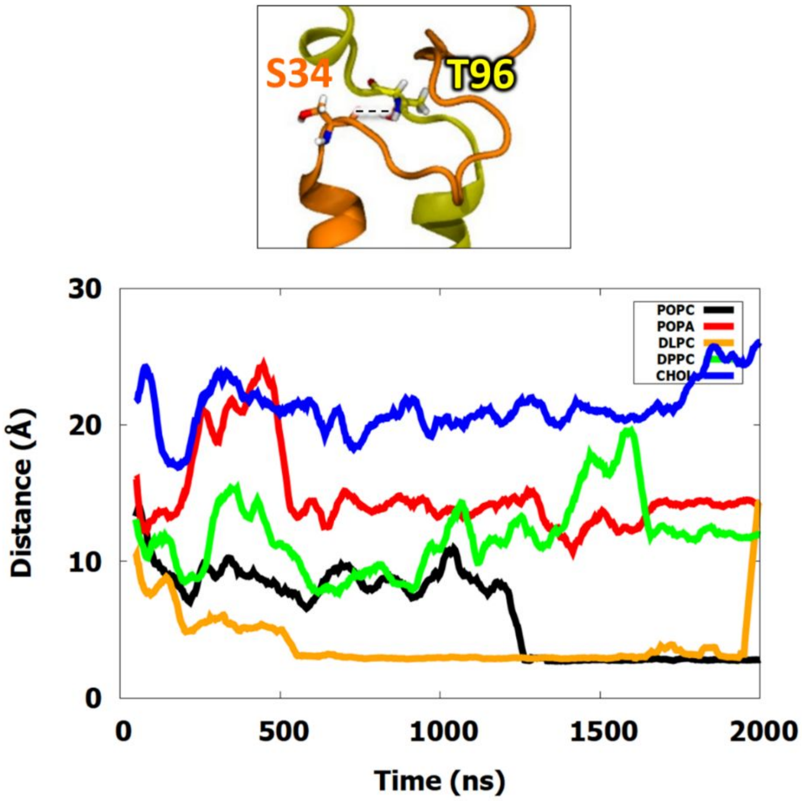

Figure S14. Distance between atoms OG@Ser34^{CD94} and OG@Thr96^{NKG2A} of the **POPC**, **POPA**, **DLPC**, **DPPC** and **CHOL** models, located at the top of the transmembrane region, indicating possible hydrogen bond formation. A moving average of 15 was used for data processing.

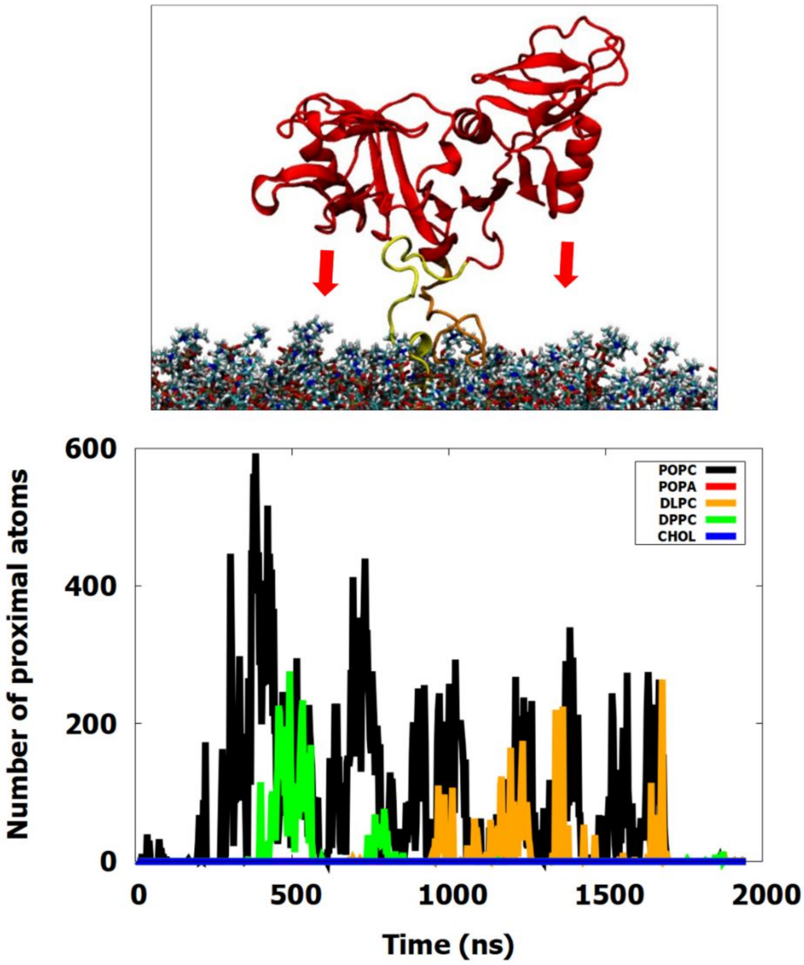

Figure S15. Total number of proximal atomic contact pairs between the extracellular domain (ECD) of NKG2A/CD94 and membrane lipids of the **POPC**, **POPA**, **DLPC**, **DPPC** and **CHOL** models. The cutoff distance was set to 4Å. The ECD of NKG2A/CD94 is shown in red.

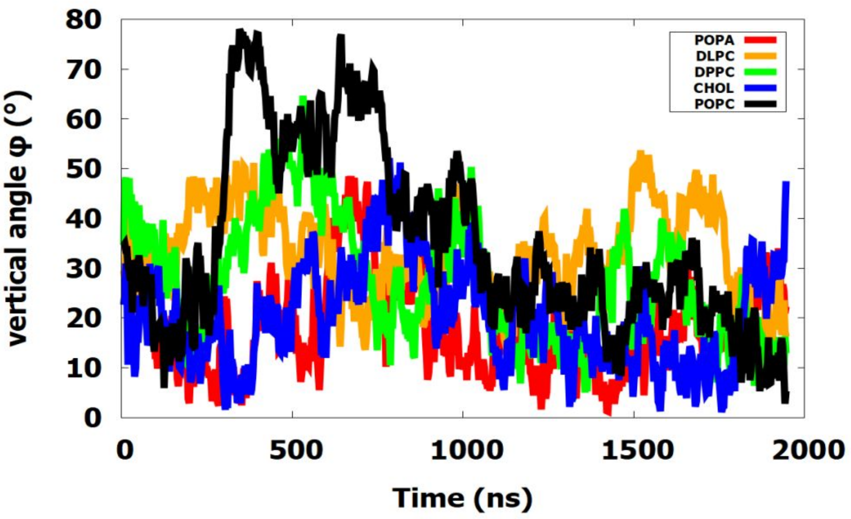

Figure S16. Vertical angle of the extracellular NKG2A/CD94 receptor domain of the **POPC**, **POPA**, **DLPC**, **DPPC** and **CHOL** models as a function of time using the vector between the centers of mass of Cys58-Ser110

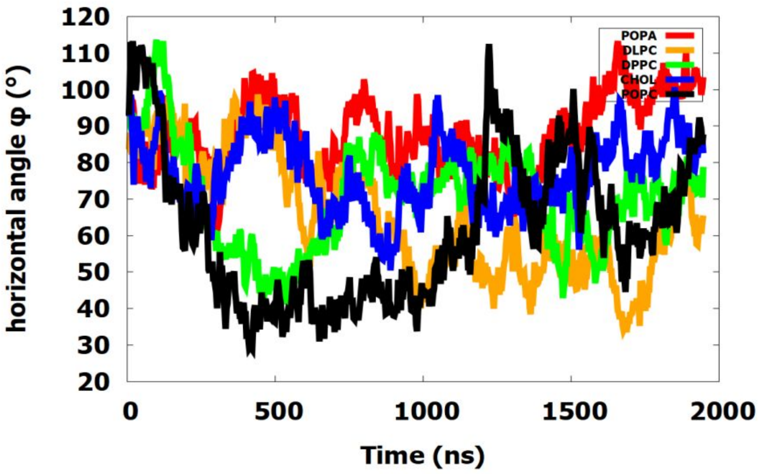

Figure S17. Horizontal angle of the extracellular NKG2A/CD94 receptor domain of the **POPC**, **POPA**, **DLPC**, **DPPC** and **CHOL** models as a function of time using the vector between the centers of mass of His184-Thr126.

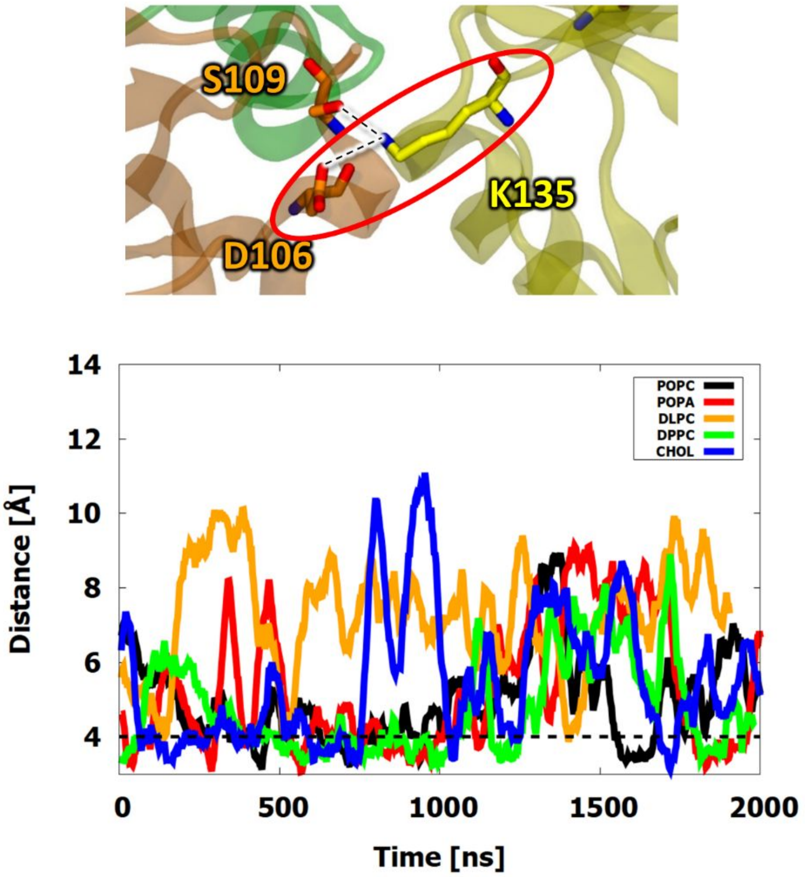

Figure S18. Distances between atoms O@Asp106 of CD94 and NZ@Lys135 of NKG2A vs. simulation time for the **POPC**, **POPA**, **DLPC**, **DPPC** and **CHOL** models. The moving average with the interval 15 was used for data processing. The interaction is circled in red in the figure.

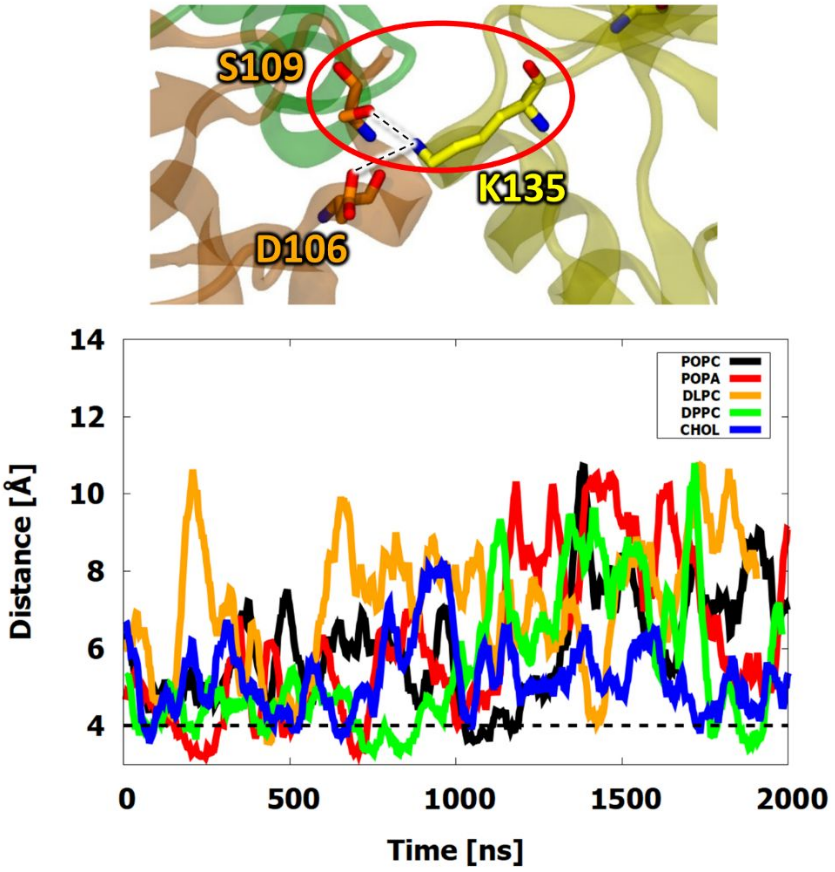

Figure S19. Distances between atoms OG@Ser109 of CD94 and NZ@Lys135 of NKG2A vs. simulation time for the **POPC**, **POPA**, **DLPC**, **DPPC** and **CHOL** models. The moving average the with interval 15 was used for data processing. The interaction is circled in red in the figure.

ANALYSIS OF MOLECULAR SIMULATIONS INCLUDING ALL REPLICAS

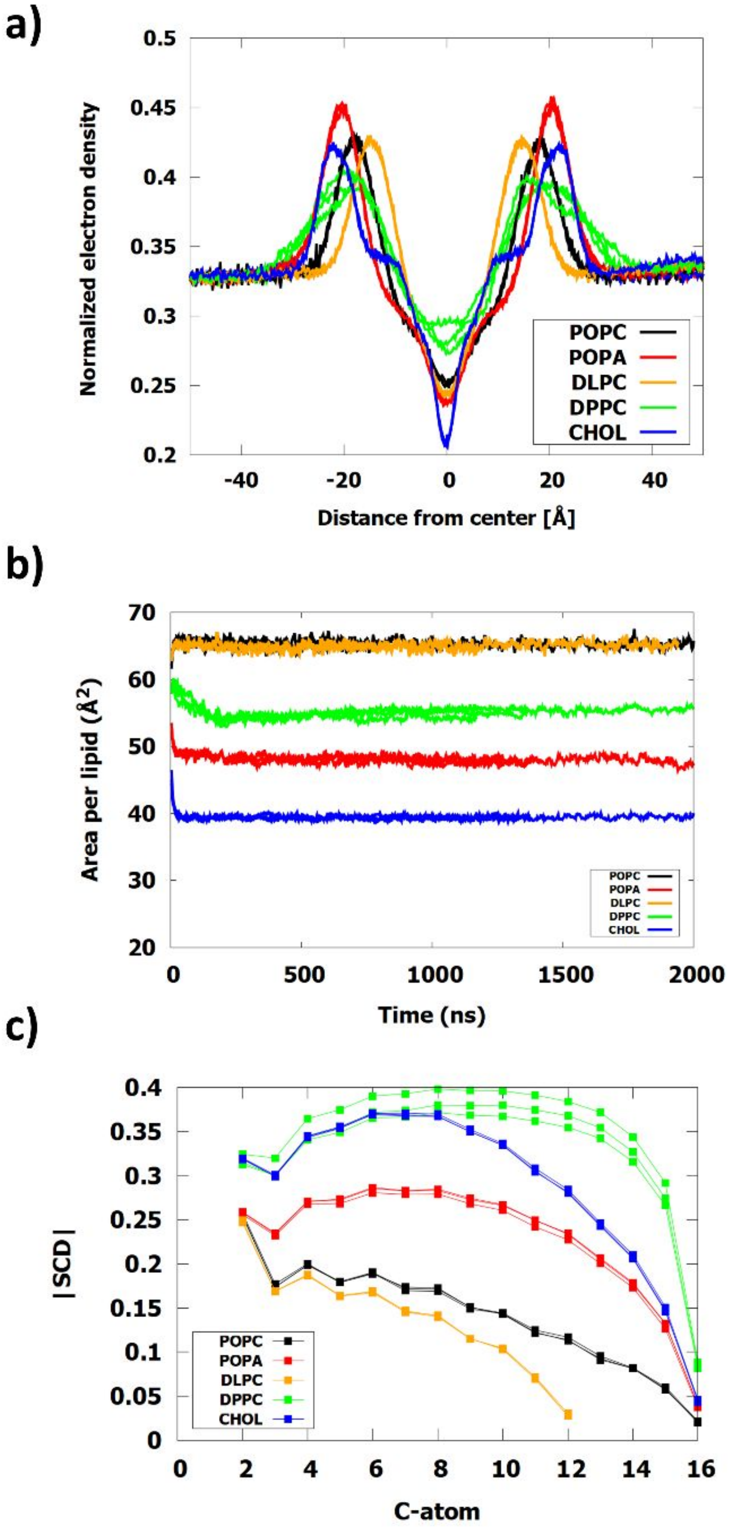

Figure S20. a) Normalized electron density profiles for all replicas of all simulated models, b) area per lipid values of all replicas and c) Absolute value of the deuterium lipid order parameters, shown as a function of the C-atom position of all replicas.

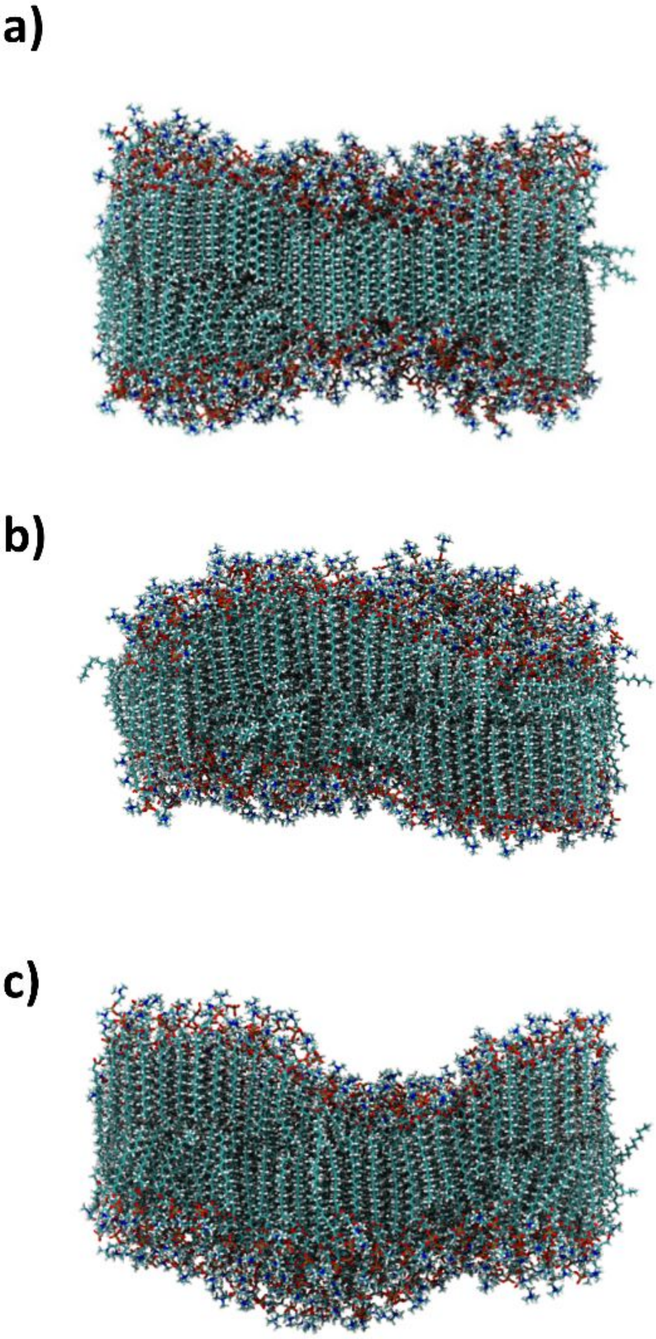

Figure S21. Membrane snapshots of **DPPC** replicas using the licorice representation at the end of the simulation trajectories of a) Replica 1, b) Replica 2 and c) Replica 3.

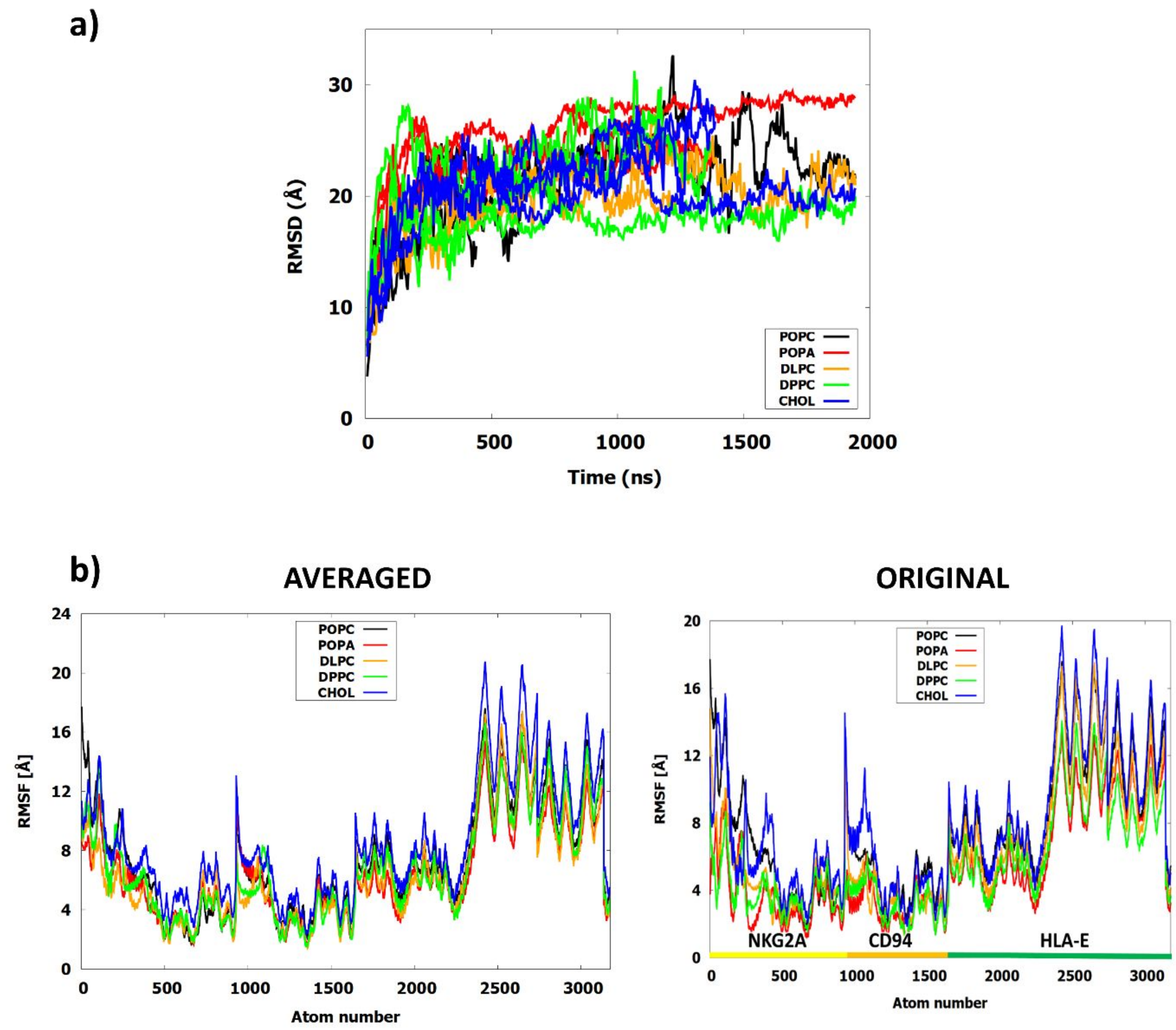

Figure S22. a) RMSD of each system including all 3 replicas and b) comparison between the averaged RMSF between all 3 replicas of each system (left) and the single replica simulations (right).

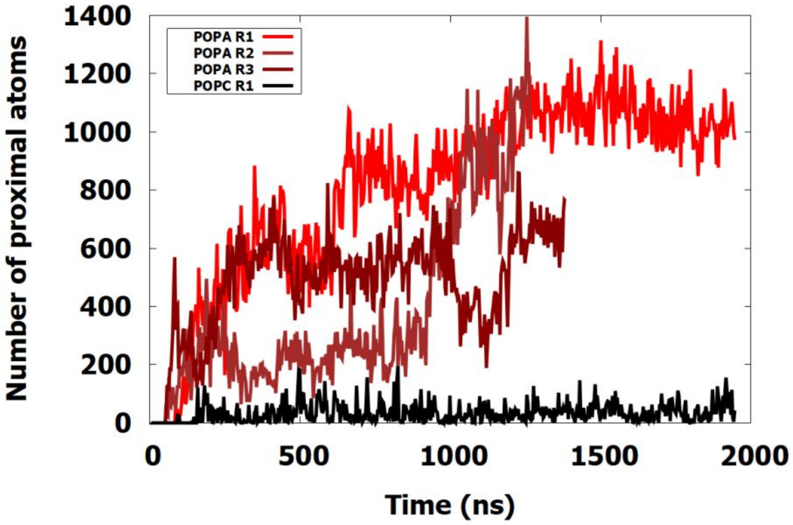

Figure S23. Total number of proximal atomic contact pairs between the IC 1-45 region of NKG2A and membrane lipids of the R1, R2 and R3 systems of **POPA,** with **POPC** for comparison. The cutoff distance was set to 4 Å.

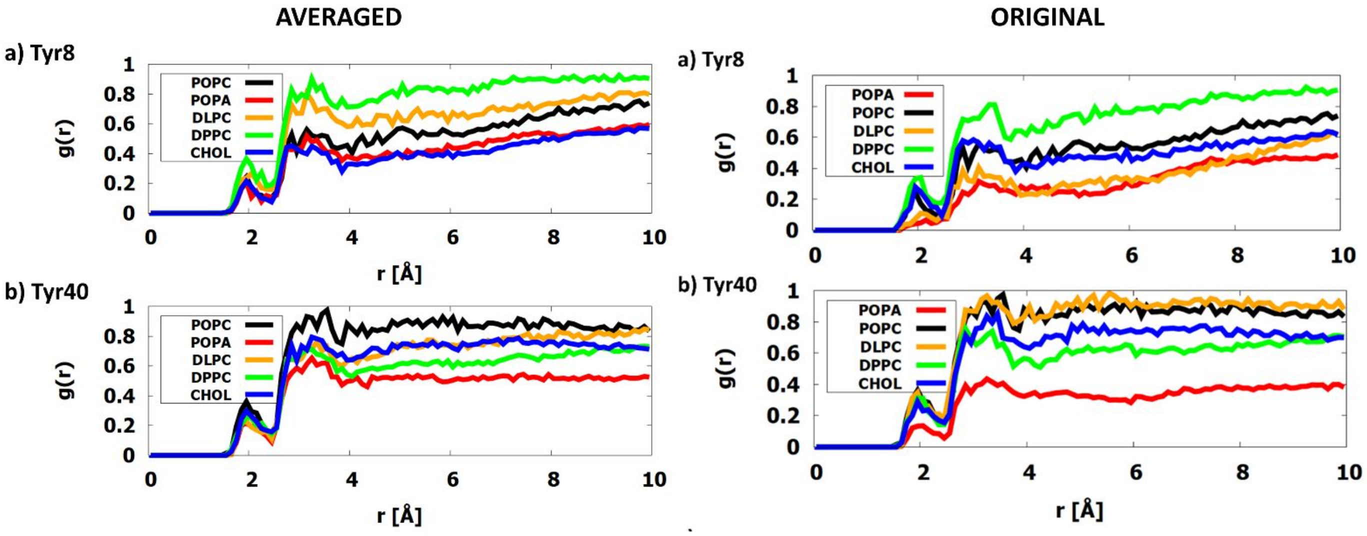

Figure S24. Comparison between the averaged RDF values between all 3 replicas of each system (left) and the initial single replica simulations (right).

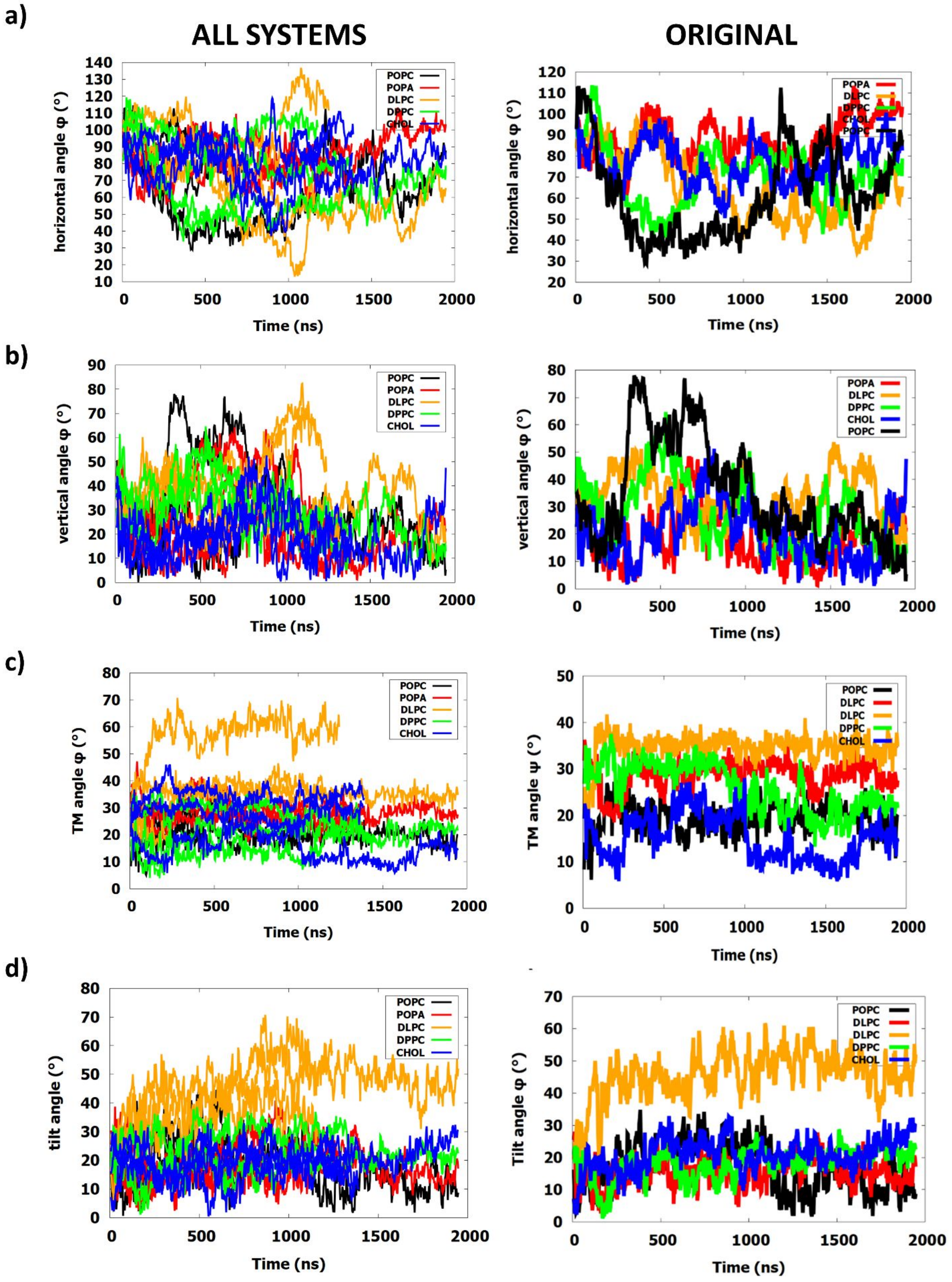

Figure S25. Angles as a function of time of all replica systems (left), with the initial single replica simulations (right) as comparison. a) horizontal ECD angle, b) vertical ECD angle, c) TM angle and d) tilt angle.

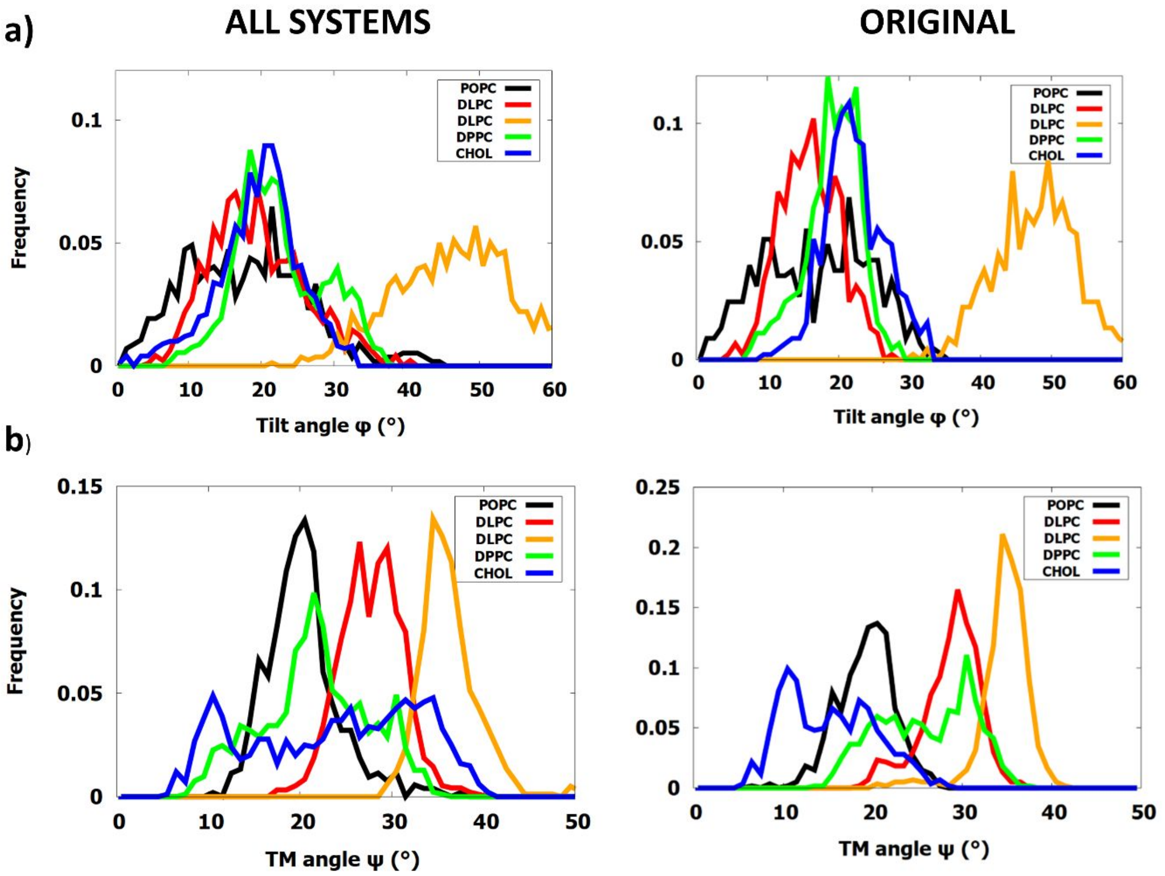

Figure S26. Comparison of histograms of a) tilt angles and b) TM angles of combined data from all replicas (left) and the original single replica simulations (right).

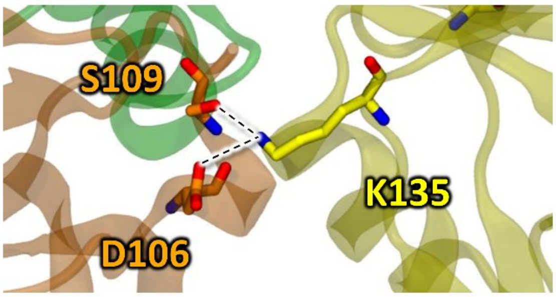

SYSTEM	Asp106^{CD94}-Lys135^{NKG2A}		Ser109^{CD94}-Lys135^{NKG2A}		Arg137^{NKG2A}-Asp149^{HLA-E}
	Persistence [%]	Distance [Å]	Persistence [%]	Distance [Å]	Distance [Å]
**R1**	7.0	7.50	13.4	7.31	9.36
**R2**	27.7	5.46	31.4	5.30	7.89
**R3**	41.0	5.17	29.8	5.90	9.12

Figure S27. Comparison of the average distance and persistence of bonds Asp106^{CD94}-Lys135^{NKG2A} and Ser109^{CD94}-Lys135^{NKG2A}, as well as the distance between Arg137^{NKG2A}-Asp149^{HLA-E} of the three **DLPC** replicas.
